# Tomato flowering depends on overlapping functions of *AP1/FUL*‐like genes in reproductive meristem specification

**DOI:** 10.1111/nph.70451

**Published:** 2025-08-19

**Authors:** Xiaobing Jiang, Iris E. Zahn, Kai Thoris, Chris Roelofsen, Edelin Roque, Concepción Gómez‐Mena, Cristina Ferrándiz, Hongru Wang, Gerco C. Angenent, Marian Bemer

**Affiliations:** ^1^ Laboratory of Molecular Biology, Wageningen University & Research Droevendaalsesteeg 1 6708 PB Wageningen the Netherlands; ^2^ Business Unit Bioscience, Wageningen University & Research Droevendaalsesteeg 1 6708 PB Wageningen The Netherlands; ^3^ Shenzhen Branch, Guangdong Laboratory of Lingnan Modern Agriculture, Key Laboratory of Synthetic Biology, Ministry of Agriculture and Rural Affairs, Agricultural Genomics Institute at Shenzhen, Chinese Academy of Agricultural Sciences Shenzhen 518120 China; ^4^ Cell and Developmental Biology, Wageningen University & Research Droevendaalsesteeg 1 6708 PB Wageningen the Netherlands; ^5^ Instituto de Biología Molecular y Celular de Plantas Consejo Superior de Investigaciones Científicas‐Universidad Politécnica de Valencia Valencia 46022 Spain; ^6^ Biosystematics group, Wageningen University & Research Droevendaalsesteeg 1 6708 PB Wageningen the Netherlands

**Keywords:** *AP1/FUL*‐like genes, flowering, inflorescence development, reproductive meristem, Tomato

## Abstract

- AP1/FUL‐clade transcription factors (TFs) are essential for the initiation and regulation of flowering and have clearly separated functions in Arabidopsis. However, how these functions have diverged across eudicots remains unclear.
- Here, we performed a detailed analysis to unravel the distinct and overlapping functions of the tomato *AP1*‐ortholog *MACROCALYX* (*MC*) and the *FUL*‐like genes *FRUITFULL2* (*FUL2*) and *MADS‐BOX PROTEIN 20* (*MBP20*) through integrated molecular, genetic, and genomic approaches.
- We find that AP1/FUL‐like TFs redundantly regulate the floral transition in both the primary shoot and sympodial shoot. In the latter, loss of *MC*, *FUL2*, and *MBP20* leads to extremely delayed flowering. In the floral and inflorescence meristem, MC is the major player, but FUL2 and MBP20 contribute as well, with a complete loss of reproductive identity in the inflorescence meristem of the triple mutant. The functional differences between the three genes can mainly be attributed to differences in expression level, as the DNA‐binding properties of MC and FUL2 are highly similar. Only the *TFL1*‐ortholog *SP* appears specifically regulated by MC.
- We reveal that the combined action of AP1/FUL‐clade TFs is needed to acquire and retain reproductive activity in tomato, which is probably conserved in many other crops.

## Introduction

The initiation of flowering and inflorescence development is essential for the reproductive success of plants and therefore controlled by extensive gene regulatory networks. The timing of the floral transition depends on a network that allows the integration of both environmental and endogenous cues by a few key regulators (Fornara *et al*., 2010). In Arabidopsis, these are FLOWERING LOCUS T (FT), the florigen that travels from the leaves to the shoot apex, and the MADS‐domain transcription factor SUPPRESSOR OF OVEREXPRESSION OF CONSTANS 1 (SOC1) (Lee *et al*., 2000; Samach *et al*., 2000; Corbesier *et al*., 2007). Subsequently, the FM identity genes *LEAFY* (*LFY*), a plant‐specific protein, and *APETALA 1* (*AP1*), which also belongs to the MADS‐domain family (Weigel *et al*., 1992; Liljegren *et al*., 1999), control the establishment of the floral meristem (FM). The activity of FM identity genes is counteracted by the PEBP‐family protein TERMINAL FLOWER 1 (TFL1), which represses *LFY* and *AP1*, and thereby FM identity, to maintain an indeterminate inflorescence meristem (IM) from which the FMs arise on the flank (Shannon & Meeks‐Wagner, 1991; Alvarez *et al*., 1992; Liljegren *et al*., 1999).

The functions of these genes appear largely conserved in other species (e.g. for *FT*, Kojima *et al*., 2002; Lifschitz *et al*., 2006; Yan *et al*., 2006; Laurie *et al*., 2011; Cheng *et al*., 2021; Abdulla *et al*., 2024), but shifts in their expression patterns as well as in the protein–protein and protein‐DNA interactions have contributed to the large diversity that exists in flowering traits among angiosperms (Kagale *et al*., 2014; Leijten *et al*., 2018). Interestingly, genes that only have a modest function in flowering in Arabidopsis, such as the MADS‐box gene *FRUITFULL* (*FUL*), appear to play essential roles in other species (Ferrándiz *et al*., 2000; Balanzà *et al*., 2014; Jiang *et al*., 2022; Martínez‐Fernández *et al*., 2024). This illustrates that Arabidopsis is not always the optimal model species, possibly because genome multiplications in the Brassicaceae have been followed by major diversification in recent evolutionary history (Kagale *et al*., 2014).

In the context of flowering, one of the still open questions is how *AP1*‐ and *FUL*‐like genes diverged in function in the eudicots, where two duplications have resulted in three subclades, named euAP1, euFULI, and euFULII (Litt & Irish, 2003). Genes from all clades have been reported to regulate aspects of flowering in different species (Ferrándiz *et al*., 2000; Kobayashi *et al*., 2012; Morel *et al*., 2019; Jiang *et al*., 2022). In Arabidopsis, AP1 has in particular an essential role in the establishment of FM identity, together with its Brassicaceae‐specific paralog CAULIFLOWER (CAL) (both euAP1‐subclade), and *AP1* is additionally considered an A‐class gene important for sepal and petal identity. FUL, on the other hand, belongs to the euFULI‐subclade and has a more minor role in the timing of the floral transition together with SOC1 (Ferrándiz *et al*., 2000; Melzer *et al*., 2008) and in the regulation of FM identity together with AP1 and CAL (Ferrándiz *et al*., 2000). Besides its role in flowering, FUL has an important function in Arabidopsis silique development (Gu *et al*., 1998; Bemer *et al*., 2017). The euFULII‐subclade gene in Arabidopsis is called *AGAMOUS‐LIKE 79* (*AGL79*), a gene that is very weakly expressed (Parenicova *et al*., 2003), not capable of dimerization (de Folter *et al*., 2005), and under relaxed constraints. Because the euAP1 and euFUL clades split at the base of the core eudicots, the functions of the *AP1*‐ and *FUL*‐like genes have likely diverged in the different eudicot lineages. Indeed, while both genes have largely specialized in Arabidopsis, with a prominent role for *AP1* in FM specification and for *FUL* in fruit development, studies in other species show a more prominent role for the euFULII‐clade gene, for example, in *Medicago truncatula* (Cheng *et al*., 2018) and pea (Berbel *et al*., 2012), or more overlapping roles for the *AP1/FUL*‐clade genes, such as in petunia (Morel *et al*., 2019).

In tomato, the euAP1‐clade gene *MACROCALYX* (*MC*), the euFULI‐clade genes *FUL1* and *FUL2*, and the euFULII‐clade gene *MBP20* all function in the regulation of flowering (Yuste‐Lisbona *et al*., 2016; Jiang *et al*., 2022), a process very different from that in Arabidopsis. Another euFULII‐clade gene, *MBP10*, may become a pseudogene due to its weak overall expression and loss of putative TF binding sites (Maheepala *et al*., 2019; Jiang *et al*., 2022). Upon the floral transition in both the primary and sympodial tomato shoot, the vegetative shoot apical meristem (SAM) differentiates into a transition meristem (TM), which further develops into a determinate FM. However, before the FM initiates floral organs, a new meristem arises on its flank and adopts IM fate. This IM follows a similar developmental trajectory as the TM, resulting in sequential FM‐IM maturations, generating a zigzagged inflorescence with multiple flowers (Lippman *et al*., 2008). In recent years, it has been shown that orthologs of all major players from Arabidopsis, SOC1, FT, AP1, LFY, and UNUSUAL FLORAL ORGANS (UFO), also regulate tomato flowering (Lifschitz *et al*., 2006; Zahn *et al*., 2023;Allen & Sussex, 1996; Molinero‐Rosales *et al*., 1999; Lippman *et al*., 2008), but there are also differences, such as the prominent role for the *WOX9* ortholog *COMPOUND INFLORESCENCE* (*S*) (Lippman *et al*., 2008), and the BTB/POZ‐domain protein TERMINATING FLOWER (TMF) (MacAlister *et al*., 2012; Xu *et al*., 2016). Also, the *TFL1*‐ortholog *SELF PRUNING* (*SP*) does not appear to play a major role in tomato inflorescence formation but acts in the maintenance of vegetative growth in the sympodial shoots (Pnueli *et al*., 1998; Shalit *et al*., 2009; Park *et al*., 2014).

In particular, the contributions of MADS‐domain proteins seem to have evolved differently than in Arabidopsis. *SOC1* co‐orthologs play a less essential role in the initiation of flowering (Zahn *et al*., 2023), while *FUL*‐like genes are more important for this initiation and additionally contribute to FM maturation (Jiang *et al*., 2022). Moreover, the *SHORT VEGETATIVE PHASE* (*SVP*)‐ortholog *JOINTLESS* (*J*) and the *AP1*‐ortholog *MC* are both essential to maintain reproductive fate in the emerging IMs (Molinero‐Rosales *et al*., 2004; Szymkowiak & Irish, 2006; Nakano *et al*., 2012; Yuste‐Lisbona *et al*., 2016). Although the *AP1* and *FUL* orthologs appear to have shifted functions compared to Arabidopsis, it is unclear to what extent these transcription factors together control the regulation of the transition to flowering in the primary and sympodial shoots, FM meristem development, and IM meristem fate, and how they are executing these functions.

In this study, we therefore investigated the distinct and overlapping functions of the AP1/FUL‐like transcription factors in tomato. We analyzed the spatiotemporal expression patterns of these genes in reproductive meristems and their protein–protein interaction profiles with MADS‐domain proteins. Through CRISPR/Cas9 mutagenesis, we examined the roles of MC, FUL2, and MBP20 in regulating flowering and inflorescence development. Additionally, we identified target genes associated with these functions by integrating qPCR, RNA‐seq, and DAP‐seq data. This revealed novel targets that are probably involved in the regulation of meristem identity, including homologs of *KINASE‐INDUCIBLE DOMAIN INTERACTING 9* (*KIX9*), *WRINKLED3/4* (*WRI3/4*) and WRKY28. Our findings suggest that the functional separation observed in Arabidopsis *AP1/FUL‐*like genes is not universal across angiosperms, but rather, the combined action of these genes is essential for flowering and maintaining reproductive activity in tomato. However, MC appears to have an additional functionality in the suppression of *SP*.

## Materials and Methods

### Plant materials and growing conditions

*Solanum lycopersicum* L. cultivar Moneyberg (a TMV‐resistant version of Moneymaker, van Rengs *et al*., 2022) was used in all experiments. Seeds were germinated either on ½ MS for tissue culture or on filter paper in trays with 50 ml water for genotyping. Tissue culture transformation was conducted in a growth chamber with 16 h : 8 h, light : dark at 25°C. Seedlings from tissue culture and seed germination were transplanted in rockwool plugs (Grodan, Roermond, the Netherlands) and cultivated in another growth chamber (16 h : 8 h, light : dark at 21°C) for *c*. 4 wk. Finally, plants were transplanted to the glasshouse of Unifarm, Wageningen University & Research, and grown under natural light supplemented with artificial light if needed. Side shoots were removed once a week, and flowers were pollinated by vibrating each flower/truss two to three times a week with an electric toothbrush.

### Yeast two‐hybrid (Y2H)

Protein–protein interaction assays were performed using the GAL4 System as described (De Folter & Immink, 2011). In short, full‐length coding sequences of *MC* and *LIN* were amplified from meristem cDNA and cloned into pDONR201 and subsequently into the pDEST32 and pDEST22 destination vectors using the Gateway™ system. The vectors for the other MADS‐domain proteins had been previously generated (Jiang *et al*., 2022; Zahn *et al*., 2023). The expression vectors were transformed into the PJ69‐4A (pDEST22) and PJ69‐4α (pDEST32) yeast strains, and the different combinations were acquired by mating. Protein–protein interactions were screened on ‐LWH dropout medium, supplemented with 3 mM 3‐amino‐1,2,4‐triazole (3‐AT), and on ‐LWA dropout medium. Plates were incubated for 5 d at 20°C. All primers used for cloning are listed in Supporting Information Table S1.

### CRISPR/Cas9 genome editing and plant transformation

The construct to create mutations in the *MC* gene using CRISPR/Cas9 was generated by GoldenGate cloning and the MoClo toolkit according to Weber *et al*. (2011). In brief, the online tool http://www.rgenome.net/cas‐designer (Park *et al*., 2015) was used for sgRNA design. Two sgRNAs were fused to the synthetic U6 promoter as U6p:sgRNA and ligated in level 1 constructs pICH47761 and pICH47772. The level 1 constructs pICH47732‐NOSpro:NPTII:OCST, pICH47742‐35S:Cas9:NOST, pICH47751‐35S:GFP:ter35S, pICH47761‐sgRNA1, pICH47772‐sgRNA2, and the linker pICH41800 were cut/ligated into the level 2 vector pICSL4723 as described in Jiang *et al*. (2022). The level 2 construct was transformed into *Agrobacterium tumefaciens* C58C1 and further transformed into Moneyberg and the *ful2 mbp20* mutant (previously generated by Jiang *et al*. (2022)) through *Agrobacterium tumefaciens*‐mediated transformation (Van Roekel *et al*., 1993). Cas9‐free T1 plants were selected by PCR for phenotyping. All primers are listed in Table S1.

### Phenotyping

Each genotype was represented by at least 10 biological replicates (plants) in the glasshouse. The primary floral transition was determined by the number of leaves below the first inflorescence, and the sympodial flowering time by the number of leaves in at least seven successive sympodial units. The inflorescence architecture was characterized after it had fully developed.

### GUS assay

The GFP‐GUS reporter constructs were cloned using the standard Golden Gate cloning strategy. In the case of *MC*, the selected promoter fragment included most of the 5' UTR and stretched until the next upstream gene (MADS‐RIN), including its 3' UTR (*c*. 3.3 kb). In the case of *FUL2*, we used a 5 kb fragment encompassing all conserved regions we identified with MEME and mVista (primers are listed in Table S1). The promoter fragments were amplified from genomic DNA and cloned into MoClo level 1 vector pICH47742. They were then combined with pICH47732‐NPTII and pICH47751‐GFP/GUS into pICSL4723 (Weber *et al*., 2011). The reporter constructs were transformed to tomato cultivar Moneyberg as described in Van Roekel *et al*. (1993). The GUS staining buffer for histochemical analysis contained 10 mM EDTA, 0.1% v/v Triton X‐100, 2 mM potassium ferricyanide, 2 mM potassium ferrocyanide, and 2 mg ml^−1^ 5‐bromo‐4‐chloro‐3‐indolyl‐beta‐d‐glucuronic acid (X‐gluc) in 50 mM phosphate buffer (pH 7.0). Tissue was infiltrated with the GUS buffer in a vacuum pump for 2 × 5 min, then incubated at 37°C overnight and immersed in 70% ethanol to remove chlorofyl. Representative samples were imaged as described below.

### RNA *in situ* hybridization

*In situ* hybridization was conducted following the protocol as described in Gómez‐Mena & Roque (2018). Briefly, reproductive meristems from WT shoot apices were dissected and fixed in FAE (3.7% formaldehyde; 5% acetic acid; 50% ethanol; v/v), cleared in Histoclear, and embedded in paraffin for sectioning. The paraffin blocks were then sectioned at 8 μm. For specificity, probe regions were designed from the 5' and 3' cDNA sequences of *FUL1*, *FUL2*, and *MBP20*, while for *MC*, only a 3' fragment was used. These fragments were amplified, cloned into the pGEM‐T vector, and transcribed *in vitro* using T7 and SP6 RNA polymerase, with Digoxigenin labeling. Both probes were then mixed for the *in situ* assay. Primer sequences for probe synthesis are listed in Table S1.

### Meristem imaging

Shoot apices of young plants were dissected using a forceps, and older leaf primordia were removed to expose the meristems for imaging under the stereomicroscope (Stemi 508; Zeiss) with a coupled camera (AxioCam IC; Zeiss). Live meristems were imaged immediately after dissection, and stained meristems were imaged after removal of Chl using incubation in 70% EtOH.

### RT‐qPCR

For RT‐qPCR analysis of gene expression, a batch of plants was grown as a biological replicate, and the first sympodial shoot vegetative meristems (SVMs) of WT, *ful2/mbp20*, and *mc/ful2/mbp20* plants were harvested. At least 30 meristems were collected for one sample using a stereomicroscope (Stemi 508; Zeiss). FM and IM were collected together to validate DEGs in FM and IM. For floral bud sampling, at least 5 small buds (*c*. 0.5–1 cm in length) were pooled as a sample; RNA was isolated by a CTAB/LiCl method (Porebski *et al*., 1997). Three batches of plants of each genotype were grown in the glasshouse consecutively for triplicate sampling. During meristem sampling, an acetone fixation technique was used to stabilize the RNA (Park *et al*., 2012), and RNA was extracted using the PicoPure RNA Extraction kit (Arcturus/Thermofisher, Landsmeer, the Netherlands). After DNase treatment with Ambion Turbo DNase (AM1907), cDNA was synthesized with the iScript cDNA synthesis kit (Bio‐Rad). Real‐time RT‐PCR was performed with the iQ SYBR Green Supermix from Bio‐Rad with a standard 2‐step program of 40 cycles, annealing at 60°C. Some primers were derived from previous work (Jiang *et al*., 2022), while the remaining primers are listed in Table S1.

### RNA‐seq

The developmental stage of the first sympodial inflorescence was visually scored under a stereomicroscope. Before the visible formation of any floral organ primordia, the first FM and IM of the inflorescence were hand‐dissected and separated using a needle. Meristems were directly frozen in liquid nitrogen after dissection and pooled before RNA extraction. RNA was extracted using the PicoPure RNA Extraction kit (Arcturus). Samples were prepared in quadruplicate, meaning that plants were grown in 4 consecutive weeks and meristems were harvested when the plants had reached the developmental stage of interest. The samples FM *mc*, FM *ful2 mbp20*, and IM *ful2 mbp20* did not contain sufficient meristems in one batch and were analyzed in triplicate. Each biological replicate contained 12–34 meristems harvested from the same batch of plants, yielding 0.4–1.9 μg total RNA.

RNA‐Seq was performed by BGI Genomics using 2 × 150 bp paired‐end DNB‐Sequencing after quality control with an Agilent 2100 Bioanalyzer. Reads were filtered by removing adaptor sequences, contamination and low‐quality reads, and mapped to the tomato SL4.0 genome using HISAT2 (Kim *et al*., 2019) with default parameters. DEGs were calculated using DESeq2 (Love *et al*., 2014), either with a Wald test when comparing two groups (FM vs IM, or WT vs mutant), or with a likelihood ratio test (LRT) when comparing more than two groups. The PCA was performed with the built‐in R package prcomp using the top 500 DEGs with most variance.

### DAP‐seq

The method was carried out as previously detailed (Bartlett *et al*., 2017; Lai *et al*., 2020) with slight modifications. The experiment focused on the heterodimers/tetramers of interest: FUL2‐J, FUL2‐J2, FUL2‐TM3, MC‐J, MC‐J2, and MC‐TM3. Additionally, a control sample, generated using TnT reaction mixture without plasmid addition, was included to account for background signals and named ‘input’. Proteins were expressed *in vitro* using the TnT®SP6 High‐Yield Wheat Germ Protein Expression System (L3260; Promega) following the manufacturer's guidelines. All proteins were cloned into a pSPUTK (Stratagene, Amsterdam, the Netherlands) vector, with a 3×FLAG tag attached to FUL2 and MC. An equimolar ratio of plasmid (up to a total of 2 μg) was used for protein mixtures. Subsequent steps were conducted at RT, using DNA LoBind® tubes (0030108051; Eppendorf, Nijmegen, the Netherlands) to enhance sample recovery. The *in vitro* produced proteins (48 μl) and DNA library from tomato leaves (400 ng) were incubated in a total volume of 360 μl EMSA binding mix for 2 h to ensure optimal protein/DNA binding conditions (Smaczniak *et al*., 2012). The binding reaction was then added to 20 μl of washed anti‐FLAG® magnetic beads (M8823; Sigma‐Aldrich), and the volume was adjusted to 1 ml by adding lysis buffer (130‐091‐125; Miltenyi Biotec, Leiden, the Netherlands) containing protease inhibitor (11 697 498 001; Roche). This mixture was incubated for 2 h on a tube revolver rotator (88 881 001; Thermo Scientific, Breda, the Netherlands), followed by three washes with 400 μl TBS. Bound proteins were eluted from the beads by incubating with 400 μl TBS containing 150 ng μl^−1^ FLAG peptides (APExBIO A6001) for 45 min on a tube revolver. Then, the supernatant was collected on a magnetic stand. A second elution step was similarly performed to obtain a total volume of 800 μl. The supernatant was incubated at 95°C for 10 min, then immediately subjected to column purification (MACHEREY‐NAGEL 740609.50S) and eluted with 50 μl Elution Buffer. The eluted DNA fragments were amplified for 20 cycles with Q5® High‐Fidelity DNA Polymerase (M0491; NEB/BIOKE, Leiden, the Netherlands) using Illumina TruSeq adaptors carrying unique barcodes. The amplified fragments were purified with AMPure XP beads (A63880; Beckman Coulter, Woerden, the Netherlands). Samples with different barcodes were pooled in equimolar ratios and sequenced with NovaSeq (Novogene, Amsterdam, the Netherlands) for 150 cycles using paired‐end sequencing. Each sample condition, run in triplicate, yielded *c*. 10–30 million reads. The reads were trimmed with Trim Galore (https://github.com/FelixKrueger/TrimGalore) and mapped to the Moneyberg genome (van Rengs *et al*., 2022) with hisat2 (Kim *et al*., 2019). For each sample, peaks were called using MACS2 (Zhang *et al*., 2008) with a significance threshold of *P* < 0.0001, and BAM files are used for differential peak analysis with MACS2 bdgdiff (Zhang *et al*., 2008). The peaks were then annotated with ChiPseeker (Yu *et al*., 2015). To obtain motif files, we performed additional peak analysis with MEME (Bailey *et al*., 2015) and GEM (Guo *et al*., 2012). The IGV genome browser (Thorvaldsdóttir *et al*., 2013) was used to visualize all peaks and sequencing data. CArG‐box spacing analysis was performed as described by Lai *et al*. (2020). For the ampDAP‐qPCR, the DAP library was amplified according to O'Malley *et al*. (2016) and an aliquot was used for qPCR (primers in Table S1). *C*_t_ enrichment values were corrected based on the primer efficiencies.

### EMSAs

Electrophoretic mobility shift assays (EMSAs) were performed as described by Smaczniak *et al*. (2012) with minor modifications. The MC coding sequence was amplified from WT Moneyberg cDNA and cloned into pSPUTK (see Table S1 for primer sequences). The FUL2 and J coding sequences were cloned into pSPUTK as previously described (Jiang *et al*., 2022). The pSPUTK promoter facilitated *in vitro* protein synthesis using the TnT SP6 High‐Yield Wheat Germ Protein Expression System (Promega), following the manufacturer's instructions. Probe fragments, ranging from 80 to 150 bp and containing the CArG‐box centrally located, were amplified from genomic DNA. Oligonucleotides were fluorescently labelled with DY‐682 via PCR using vector‐specific DY‐682‐labeled primers, followed by purification with the NucleoSpin Gel and PCR Clean‐up Kit (Macherey‐Nagel, Dueren, Germany). Gel shifts were visualized using a LiCor Odyssey imaging system at 700 nm.

## Results

### The tomato AP1/FUL‐orthologs share functions in the reproductive meristems

**Fig. 1:**
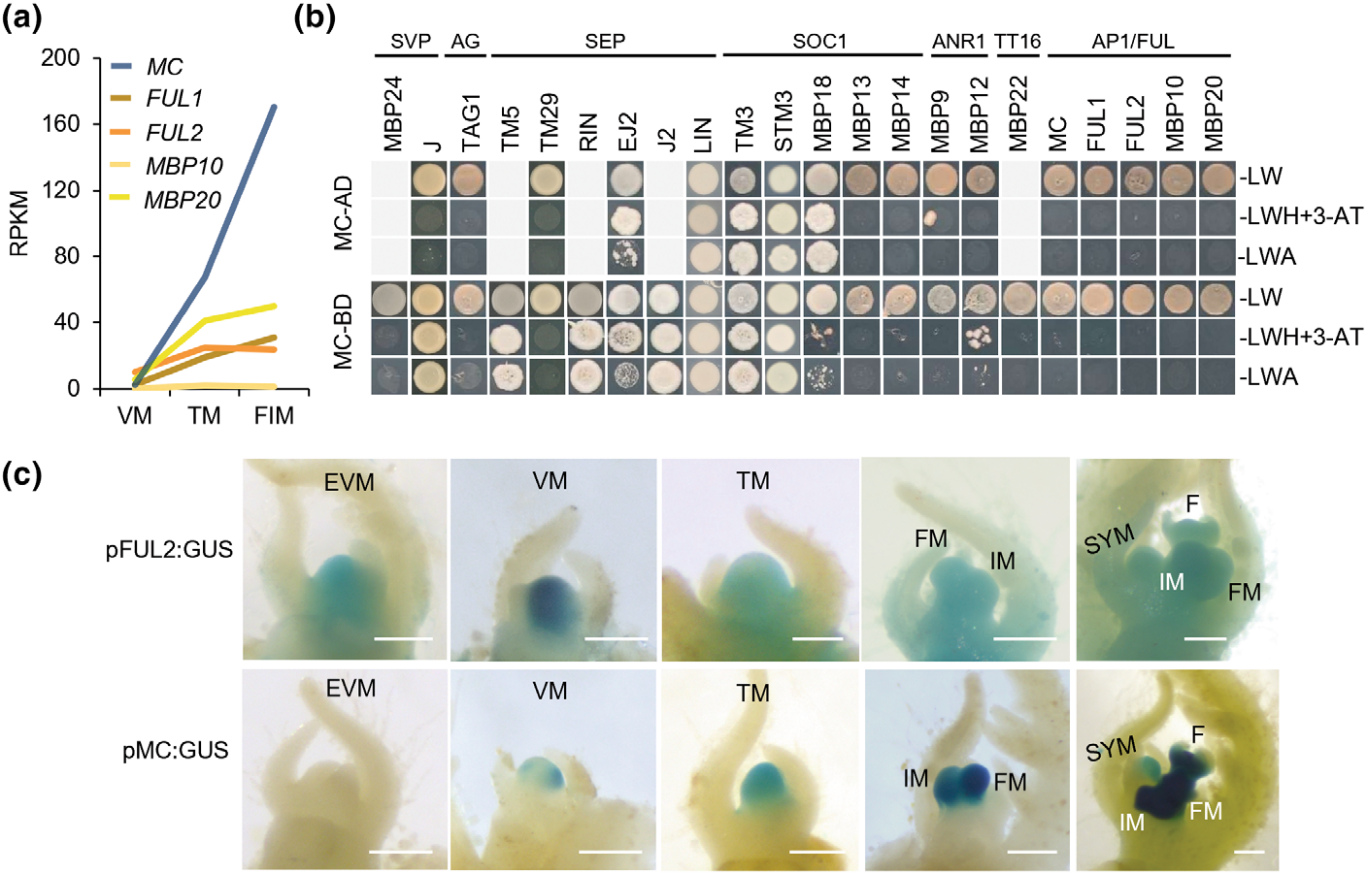
Characterization of the tomato AP1/FUL‐like MADS‐box genes/proteins. (a) Normalized gene expression (RPKM) of *AP1/FUL*‐like genes in the primary shoot meristem of wild‐type. The values shown are the average of three replicates. FIM, floral meristem and inflorescence meristem; TM, transition meristem; VM, vegetative meristem. (b) Protein interactions between the MC protein and other tomato MADS‐domain proteins in a yeast 2‐hybrid assay. Grey boxes indicate that the interaction was not tested, in most cases, because the bait gave auto‐activation. A, adenine; H, histidine; L, leucine; W, tryptophan; 3‐AT, 3‐amino‐1,2,4‐triazole (tested concentration: 3 mM 3‐AT). (c) GUS staining of pFUL2:GUS and pMC:GUS reporters in shoot apical meristems. EVM, early vegetative meristem; F, flower bud; FM, floral meristem; IM, inflorescence meristem; SYM, sympodial meristem. White bar, 200 μm.

The M‐ and K‐ domains of AP1/FUL‐like proteins are highly conserved, while their I‐domain and C‐terminus exhibit greater divergence, suggesting potential functional diversification (Fig. S1). To determine to what extent the functions of the *AP1/FUL*‐like genes overlap during reproductive meristem development, we investigated their expression profiles in detail in existing transcriptome data (Jiang *et al*., 2022) (Fig. 1a). *MC* is lower expressed than *FUL2* and *MBP20* in the vegetative meristem (VM), at a similar level as *FUL1*, but its expression sharply increases in TM and FM, reaching a much higher level than any of the tomato *FUL*‐like genes (*SlFULs*). This expression pattern is consistent with a prominent role for *FUL2/MBP20* in flowering time regulation (Jiang *et al*., 2022), and a more important role for *MC* in FM development (Molinero‐Rosales *et al*., 2004; Szymkowiak & Irish, 2006).

To investigate the hypothesis that MC and the SlFULs act redundantly in the same protein complexes when co‐expressed, we tested the protein–protein interactions that can be formed by MC and compared them with the set that we identified before for the four FUL‐like proteins (Jiang *et al*., 2022; Zahn *et al*., 2023). Among the FUL‐like proteins, FUL2 displayed most interactions, with 11 of the 22 tested tomato MADS‐domain proteins, while FUL1 and MBP20 both interacted with a subset of these, and MBP10 hardly interacted at all (Jiang *et al*., 2022). We used the same set of MADS‐domain proteins to screen for the MC protein–protein interactions and found that MC could interact with the same proteins as FUL2, except for TAG1 and TM29, for which no interaction was observed (Figs 1b, S2 for a comparison of the MC and SlFUL interactions) (Leseberg *et al*., 2008; Jiang *et al*., 2022). Our results are consistent with earlier yeast two‐hybrid studies in which a few MC interactions were tested, with the exception of the interactions with TM5 and EJ2, which were not reported previously (Leseberg *et al*., 2008; Jiang *et al*., 2022; Zahn *et al*., 2023). Like the SlFULs, MC cannot form a homodimer, nor can it heterodimerize with any of the SlFULs. The similar protein–protein interaction patterns of MC and FUL2 hint towards overlapping biological functions.

To investigate in more detail the putative distinct and overlapping functions of *FUL2* and *MC* during reproductive meristem development, we generated pFUL2:GUS and pMC:GUS stable transgenic lines and performed a histochemical assay (Fig. 1c). The GUS stainings of pFUL2:GUS and pMC:GUS show that *FUL2* is active before *MC* in the VM, while *FUL2* and *MC* have similar expression patterns in the FM and IM, with more intense staining for *MC*. Notably, the *FUL2* signal is also present in the stem below the meristem. Together, our detailed expression analysis and protein–protein interaction studies suggest that FUL2 may be more important than MC in the primary transition to flowering, while both genes potentially have redundant functions in the IM, FM, and sympodial shoot meristem (SYM). Because *FUL1* and *MBP20* are also considerably expressed in reproductive meristems and share interaction partners with MC (Fig. 1a,b), we additionally performed *in situ* hybridizations with specific probes to assess their potential contributions. The results clearly showed that *MBP20* and *FUL1* transcripts are located in the IM, FM, and SYM (Fig. S3), and thus probably have overlapping functions. Additionally, the *in situ* hybridizations confirmed *FUL2* and *MC* expression in the FM and IM (Fig. S3), consistent with previous findings with a less specific probe (Yuste‐Lisbona *et al*., 2016).

### The tomato *AP1* /*FUL‐*like genes together regulate the transition to flowering, particularly in the sympodial shoot

**Fig. 2:**
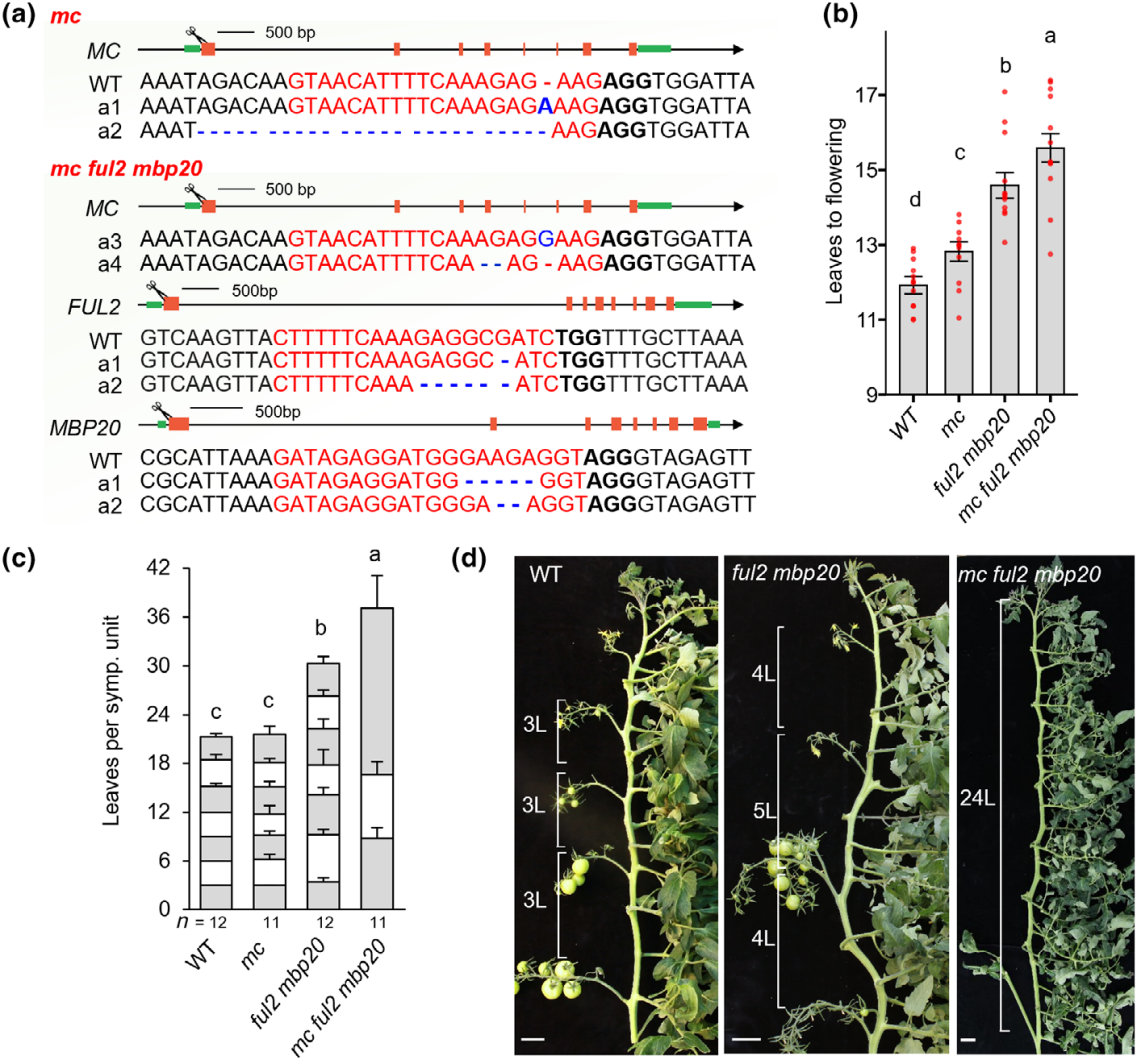
Floral transition phenotypes of the tomato *mc*, *ful2 mbp20* and *mc ful2 mbp20* mutants (a) CRISPR‐induced out‐of‐frame deletions and insertions (in blue) in the characterized lines. The red font highlights sgRNA targets with protospacer‐adjacent motif (PAM) sequences in black bold. Cartoon scissors indicate the targeted exons in the depicted gene model on top. (b) Primary shoot flowering time indicated by the number of leaves to the first inflorescence in wild‐type and mutants. Red dots indicate the value for each measured individual (c) Flowering times from the successive sympodial shoots of the same set of plants as in (b). The average leaf number of all sympodial shoots from each plant was used for statistical significance analysis. *n*, number of individual plants measured. (d) Representative main shoots from wild‐type and mutant plants. For the *mc ful2 mbp20* mutant plant, only the third sympodial unit is shown, and the third inflorescence was removed due to bushy vegetative growth. L, leaf. Bars, 5 cm. In (b, c), mean values (± SD) were compared between genotypes using one‐way ANOVA followed by a *post hoc* LSD test; different letters indicate the significance at *P* < 0.05.

To investigate the unique and redundant functions of *MC* and the *SlFUL*s *in planta*, we knocked out *MC* in the wild‐type (WT) as well as in the *ful2 mbp20* mutant (Jiang *et al*., 2022) (Fig. 2a). The *ful2 mbp20* double mutant was selected because *FUL2* and *MBP20* have previously been shown to regulate both the floral transition and inflorescence architecture in a redundant manner (Jiang *et al*., 2022). We confirmed the previously observed one‐leaf delay in flowering in primary shoots of the *mc* mutant (Yuste‐Lisbona *et al*., 2016). Additionally, we observed that the *mc* mutation enhanced the *ful2 mbp20* flowering phenotype (Fig. 2b). The delayed flowering is condition‐dependent and was much greater in an autumn glasshouse trial than in spring (Figs 2b, S4). The stronger flowering phenotype of *ful2 mbp20* compared to *mc* indicates a larger contribution of *FUL2* and *MBP20* to the primary floral transition. In sympodial shoots, however, which in wild‐type and *mc* single mutants make the transition to flowering after three leaves (Fig. 2c,d), the combined loss of *MC, FUL2*, and *MBP20* dramatically delayed sympodial flowering. While *ful2 mbp20* sympodial shoots flower after 4–5 leaves, *mc ful2 mbp20* shoots remain in a vegetative state progressively longer, with even a 24‐leaf floral transition in the third sympodial shoot, after which flowering was abolished. This phenotype was variable as well, and even more severe outside the growing season (autumn/winter). These results indicate that *FUL2/MBP20* are most important for flowering time in both the primary and sympodial shoots. However, *MC* also contributes, particularly in the sympodial shoots, where the *AP1/FUL*‐like genes turn out to be major determinants of the floral transition.

### *mc ful2 mbp20* mutants show enhanced inflorescence defects

**Fig. 3:**
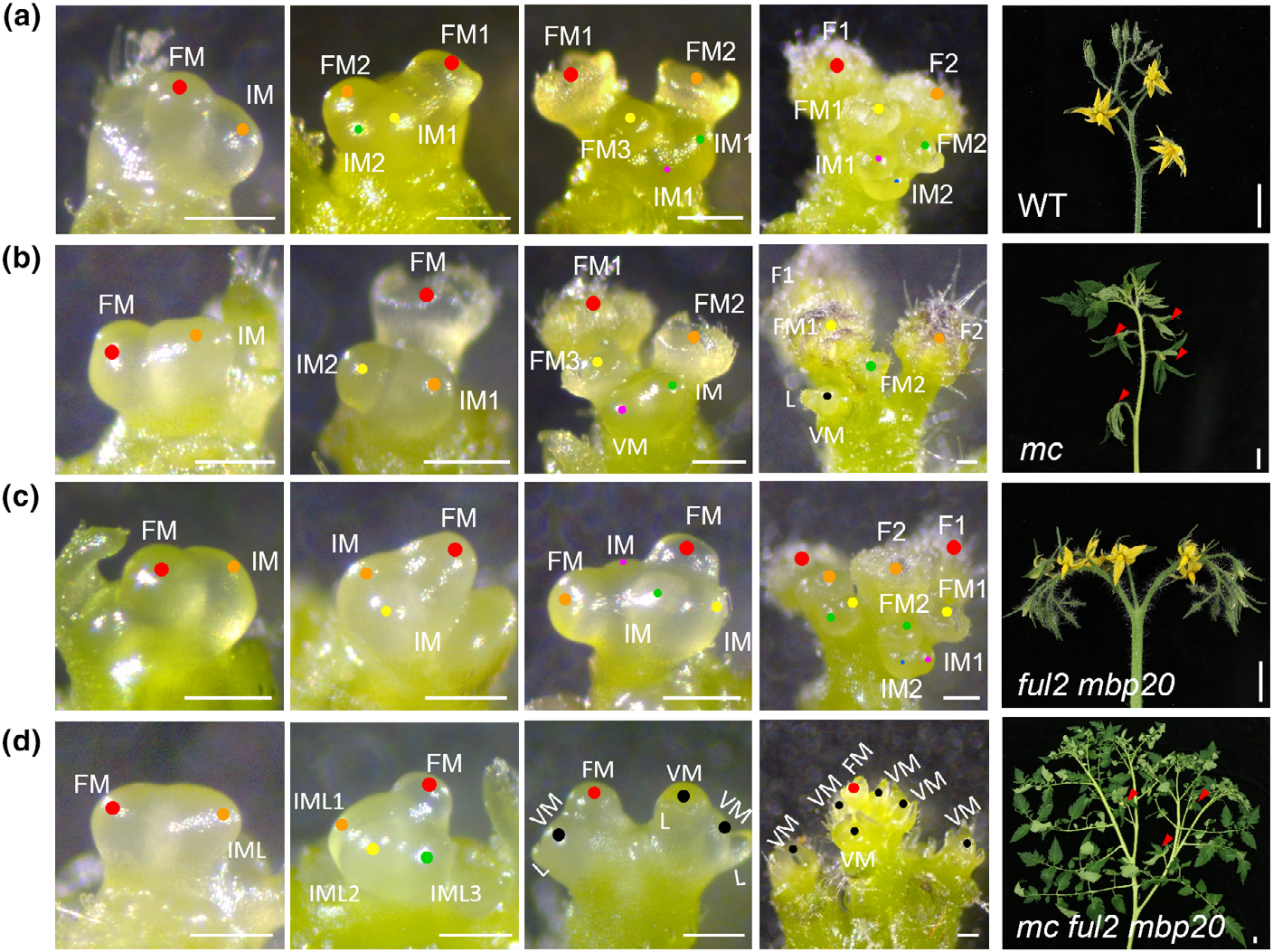
Loss of *AP1/FUL*‐like genes causes severe inflorescence defects in tomato by changing IM fate. (a–d) Developmental series of a sympodial meristem after the transition to flowering, from the FM stage to an inflorescence. (a) WT, (b) *mc* mutant, (c) *ful2 mbp20* mutant, and (d) *mc ful2 mbp20* mutant. F, flower; FM, floral meristem; IM, inflorescence meristem; IML, IM‐like; VM, vegetative meristem; L, leaf. Colored dots reflect sequential meristem initiation (order of appearance: red, yellow, orange, green, purple, and blue); black dots highlight VMs. White bar, 200 μm. The panels on the right show representative inflorescences from all genotypes. The red arrowheads indicate flowers. White bar, 2 cm.

It has previously been shown that the inflorescences of *mc* plants revert to vegetative growth after a few flowers with leaf‐like sepals have been formed (Nakano *et al*., 2012; Yuste‐Lisbona *et al*., 2016). We observed the same in our *mc* single mutants but found that *FUL2* and *MBP20* mutations severely enhanced the vegetative reversion of the *mc* mutant inflorescences (Fig. 3), with vegetative reversion directly after the formation of the first flower. The resulting bushy inflorescence made occasionally again the transition to flowering, reminiscent of sympodial shoots (Fig. 3d). To explore the developmental basis for the inflorescence defects in each genotype, we dissected and compared the SAM growth dynamics at different reproductive stages. As previously shown, *ful2 mbp20* mutant inflorescences are branching due to delayed maturation of the FM, allowing more than one IM to form (Lippman *et al*., 2008; Jiang *et al*., 2022). Branching appears to occur less in *mc* single mutants, although this phenotype is also variable, ranging from non‐branched to multiple‐branched inflorescences (Figs 3c, S5a). In *mc*, after the initiation of a few flowers, the lateral meristem adopts a vegetative fate of either leaf development or shoot growth, and this vegetative reversion occurs much earlier in *mc ful2 mbp20*, initiating only one FM that develops into a flower with leaf‐like sepals (Figs 3b,d, S5b). In *ful2 mbp20* and *mc ful2 mbp20*, the maturation of the first FM is delayed, allowing additional IM‐like (IML) meristems to form on its flank, and these IMLs adopt vegetative fate to make a bushy inflorescence. Thus, *MC* controls IM specification with a contribution of *FUL2/MBP20*. This is supported by the fact that the inflorescences of *slful* higher order mutants (*ful1 ful2 mbp20*) revert to leaf and/or shoot growth occasionally, but more often than those of WT plants (Fig. S6).

### Potential downstream targets of MC and FUL2/MBP20 in the sympodial shoot floral transition

**Fig. 4:**
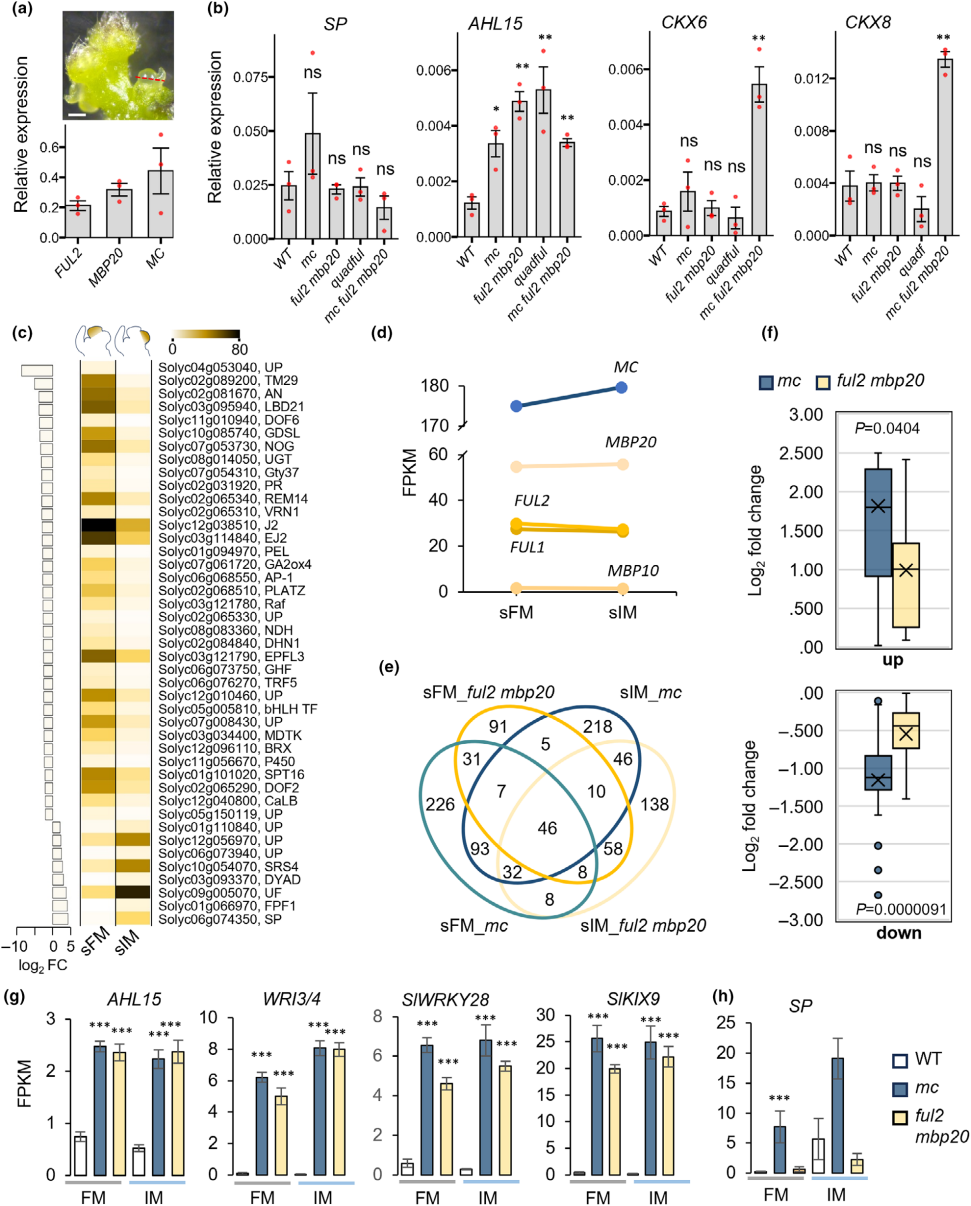
Target gene analysis in the tomato SYM, FM, and IM. (a) Upper panel: manual dissection of the SVM (sympodial shoot vegetative meristem), tissue above the red line was sampled; lower panel: the *AP1/FUL*‐like gene expression in SVM. White bar, 200 μm. (b) Gene expression in the SVM obtained by qRT‐PCR. Red dots indicate the value for each measured individual. (c) DEGs differentially expressed in FM and IM. Left bars, expression difference (log_2_ fold change) between FM and IM. The heatmap represents average FPKM values of four biological replicates. The schematic apices on top show the sampled meristems. The abbreviations for the gene names listed are derived from their respective full names. UP, unknown protein. (d) Expression of the *AP1/FUL*‐like genes in the FM and IM. (e) Venn diagram displaying the number of specific and common DEGs (*P*adj < 0.01). All genes in the Venn diagram are included in Supporting Information Dataset S2. (f) Boxplot displaying the distribution of log_2_ fold changes in gene expression for DEGs in *mc* and *ful2 mbp20* mutant FMs. The *P* value was calculated using a one‐tailed independent Student's *t*‐test. Upper plot: upregulated genes; lower plot: downregulated genes. (g) Strongly upregulated common DEGs in the FM and IM of WT, *mc*, and *ful2 mbp20* (qRT‐PCR validation in Fig. S13). (h) *SP* is specifically upregulated in *mc* mutants. The data represent mean FPKM values ± SE from three biological replicates (FM *mc*, FM *ful2 mbp20*, IM *ful2 mbp20*) or four biological replicates (FM WT, IM WT, IM *mc*). Significant differences compared to the respective WT (FM or IM) are indicated by asterisks (***, *P*_adj_ < 0.001, in (g, h)). In (b), values represent the mean ± SE of three biological replicates. Significant differences were determined using a one‐tailed Student's *t*‐test (*, *P* < 0.05; **, *P* < 0.01). ns, non‐significant.

Since sympodial shoot flowering is unaffected in *mc* mutants, mildly delayed in *ful2 mbp20* mutants, but dramatically delayed in *mc ful2 mbp20* mutants, we wondered how the three genes together regulate sympodial flowering time. Interestingly, *MC*, *FUL2*, and *MBP20* have similar expression levels in the sympodial shoot vegetative meristem (SVM) (Fig. 4a), in contrast to the primary VM. To investigate if the dramatic delay was linked to upregulation of the known sympodial cycling regulator *SP* or its interactor, SP‐interacting G‐BOX (*SPGB*) (Carmel‐Goren *et al*., 2003; Shalit *et al*., 2009; Park *et al*., 2014), we harvested SVMs from WT, *mc*, *ful2 mbp20*, *ful1 ful2 mbp10 mbp20* (*quadful*) and *mc ful2 mbp20* mutants. Harvesting sufficient SVMs from *mc ful2 mbp20* plants was very challenging, given their highly variable delay in the primary and sympodial floral transitions. We obtained three samples, which each contained several pooled SVMs, but the degree of vegetativeness of these meristems was difficult to assess. There was no significant difference in the expression levels of *SP* and *SPGB* in the different mutant backgrounds, indicating that the delayed floral transition is not regulated via the SP‐SPGB module (Fig. 4b). Therefore, we tested other genes reported to regulate the floral transition or acting downstream of the tomato *FUL*‐like genes (Jiang *et al*., 2022; Zahn *et al*., 2023; Huerga‐Fernández *et al*., 2024). The vegetative markers *APETALA 2b* (*AP2b*) and *AP2c*, involved in the primary floral transition (Zahn *et al*., 2023; Huerga‐Fernández *et al*., 2024), were not significantly dysregulated in any of the mutants, nor was the expression of *BRANCHED 1b* (*BRC1b*) (Fig. S7). However, significant differences were observed in the expression levels of a close homolog of *AT‐HOOK MOTIF CONTAINING NUCLEAR LOCALIZED 15* (*AHL15*), a meristem maturation gene (Karami *et al*., 2020; Jiang *et al*., 2022) and the cytokinin oxidase genes *CKX5*, *CKX6*, and *CKX8*, which have been previously associated with the primary floral transition (Jiang *et al*., 2022) (Figs 4b, S7). *AHL15* was derepressed in all mutant combinations, indicating that it is controlled by both MC and the SlFULs. However, surprisingly, there was no additive effect detected in the *mc ful2 mbp20* triple mutant. By contrast, all three tested *CKX* genes were only strongly upregulated in the *mc ful2 mbp20* mutant, without significant effect in any of the other mutant combinations. This could indicate that the *AP1/FUL‐like* genes act completely redundantly in the regulation of the *CKX* genes in the SVM, but it may also indicate that the *mc ful2 mbp20* SVM samples have a different (more vegetative) stage than the other harvested samples. In conclusion, the results suggest that it is not the SP‐SPGB module, but rather the combination of a few other downstream genes, such as *AHL15* and *CKX5/6/8*, that are repressed by *AP1/FUL‐like* to promote the sympodial floral transition.

### Transcriptome profiling of FMs and IMs

To further dissect the roles of MC and FUL2/MBP20 in the regulation of IM and FM identity, we performed transcriptomic profiling of WT, *mc*, and *ful2 mbp20* separated FMs and IMs. Due to the largely delayed flowering of the *mc ful2 mbp20* plants, we were unfortunately unable to harvest sufficient meristems from this mutant. We hand‐dissected the inflorescences of the first sympodial shoot (i.e. the second inflorescence) and separated the first FM and IM before the visible formation of floral organs (Fig. 4c). The first FM/IM was chosen to avoid harvesting dissimilar tissues, because after the production of a few flowers, *mc* mutant inflorescences revert to the vegetative stage. High‐throughput sequencing of these samples resulted in distinct transcriptomic profiles for both meristem types. To assess the quality of these profiles with respect to FM/IM tissue separation, and to gain insight into genes that specifically mark the FM or IM stages, we explored which meristem‐maturation markers are enriched in our FM and IM samples. For a complete time course, the generated FM/IM expression data were combined with data of the primary VM, TM, and pooled primary FM/IM from a previous study in Moneyberg (Zahn *et al*., 2023). We selected genes that have been reported as meristem‐maturation markers (Park *et al*., 2012; Lemmon *et al*., 2016; Meir *et al*., 2021) and that are dynamically expressed over the five stages of reproductive meristem development (*P*_adj_ < 0.05, 89% of reported markers). Clustering of these 2608 marker genes based on the developmental stage with the highest expression level revealed clear expression profiles for all developmental stages (Fig. S8; Dataset S1). The FM samples display high expression of the FM markers *FA* and *ANANTHA* (*AN*, ortholog of *UFO*) (Lippman *et al*., 2008), while the IM markers *UNIFLORA* (*UF*) and *LONG INFLORESCENCE* (*LIN*, ortholog of *SEP4*) peak in the IM samples (Dielen *et al*., 2004; Soyk *et al*., 2017), thereby confirming that the separation of the distinct meristems was successful. In the FM data, many specific or highly enriched genes were identified, including *J2* and *EJ2* (*SEP4* orthologs), of which the proteins interact with FUL2 and MC (Fig. 4c). Only a few specific genes were associated with the IM, namely *LIN* (Fig. S8), *SlWUS* (ortholog of *WUS*) (Fig. S8), the floral repressor *SP*, *UNIFLORA* (*UF*), the SHI‐RELATED SEQUENCE (SRS)‐family gene SlSRS4 (Lu *et al*., 2023), the GA‐signaling gene *FLOWERING PROMOTING FACTOR 1* (*FPF1*) (Lee *et al*., 2022), and three unknown proteins (Fig. 4c).

To learn more about the contributions of the *AP1/FUL*‐like genes in FM and IM regulation, we checked their expression levels in the first sympodial FM and IM and found that *MC* is highest expressed in both meristem types. Its levels are about three times higher than those of *MBP20*, which is the second highest expressed, followed by *FUL1* and *FUL2*. Finally, *MBP10* is not expressed in both meristem types (Fig. 4d). Notably, these expression values differ from those observed in the primary shoot meristems (i.e. the first inflorescence), where the expression of *FUL1* depends on FUL2/MBP20, and *MC* is weaker expressed than *FUL2/MBP20* in VM (Fig. 1a) (Jiang *et al*., 2022). The contribution of *FUL1* appears minor though, as *ful2 mbp20* and *ful1 ful2 mbp20* mutants have very similar phenotypes (Jiang *et al*., 2022). Interestingly, *MC, FUL1, FUL2*, and *MBP20* each have similar expression levels in FM and IM, suggesting that they play roles in both meristem types, in line with their capacity to interact with J2/EJ2 and LIN. The higher expression of *MC* compared to *FUL2* and *MBP20* points towards a more important function for the *AP1*‐ortholog.

### Largely overlapping target gene sets reflect the FM‐to‐IM identity shift in *mc* and *ful2 mbp20* mutants

To get more insight into the differences between the role of the *AP1*‐like gene *MC* and the *FUL*‐like genes *FUL2/MBP20* in sympodial FM and IM formation, we performed differential expression analysis. Principal component analysis (PCA) of all samples revealed that PC1, which explains 34% of the variance (Fig. S9), is associated with many of the FM or IM markers identified in Fig. 4(c), including *AN, TM5, TM29, REM14, LBD21, UF*, and *SlSRS4*. Thus, PC1 directly reflects FM/IM identity, while PC2 and PC3, which explain 13% and 11%, respectively, both appear to separate the samples based on their genotype (independent of meristem identity).

In the PCA plot, the WT FM and IM samples are extremities on the PC1 axis, while the FMs of the mutants are shifted toward the IM (Fig. S9). Especially, the *mc* FM is closer to IM than to FM identity, indicating that the *mc* FM is more similar to the IM on the transcriptome level. The *ful2 mbp20* FM is positioned between the FMs of WT and *mc*, suggesting a less important role for FUL2/MBP20 in the acquisition of FM identity. We observed that many differentially expressed genes (DEGs) displayed minor changes in expression level, probably reflecting the meristem identity shift (or FM maturation delay) rather than being a direct effect of loss of *MC/FUL2/MBP20* activity. To find a balance between the low fold changes and significance, we determined the DEG lists based on a *P*adj < 0.01, a fold change > 1.25 or < 0.8, and FPKM values > 1. For the FM samples, this resulted in 256 and 451 DEGs for *ful2 mbp20* and *mc*, respectively. The overlap between the DEGs from both mutants is 92 (Fig. 4e; Dataset S2), which is lower than we expected. Therefore, we inspected the DEG lists in more detail and discovered that of the 359 ‘specific’ *mc* DEGs, 292 showed the same expression trend in *ful2 mbp20*, albeit not significant. In line with this, 291 of the 292 genes were more strongly dysregulated in *mc* than in *ful2 mbp20* (quantified in Fig. 4f). Similarly, of the 323 specific *mc* DEGs in the IM, 214 show a comparable trend in *ful2 mbp20* (Dataset S2). Moreover, 101 genes were differentially expressed in the *mc* mutant but had WT expression levels in *ful2 mbp20*, suggesting that the dominant effect of *MC* on IM fate is even more prominent than on FM fate. Notably, although some FM markers have changed their expression in *mc* and *ful2 mbp20*, most of them, such as *AN, S, LBD21, PUCHI, DOF2, DOF6*, *J2, EJ2*, and *TM2*9, have not significantly changed (Dataset S3), indicating that the shift towards IM/TM identity depends on the cumulative expression change of only part of the IM‐ and FM‐identity genes. It is plausible that this shift causes the delayed maturation in *ful2 mbp20* and *mc ful2 mbp20* FMs, associated with branching (Fig. 3c,d).

### MC and FUL2/MBP20 repress the same genes to control reproductive meristem identity

To better understand how MC and FUL2/MBP20 regulate reproductive meristem identity, we focused on several of their DEGs, which are strongly repressed by both and encode transcription (co‐)factors (Fig. 4g; Table S2; Dataset S3). These are: *AHL15‐like* (*AT‐HOOK MOTIF NUCLEAR‐LOCALIZED PROTEIN 15*, Solyc12g087950), a suppressor of axillary meristem maturation known to be regulated by FUL in Arabidopsis (Karami *et al*., 2020), *WRI3/4*‐like (Solyc06g068570), an AP2/ERF TF that regulates fatty acid accumulation and ABA response in Arabidopsis (Lee *et al*., 2009; To *et al*., 2012), a WRKY TF (*SlWRKY28‐like*, Solyc12g011200), a regulator of cell fate and leaf senescence in Arabidopsis (Zhao *et al*., 2018; Tian *et al*., 2020), and *SlKIX9*‐like (*KINASE‐INDUCIBLE DOMAIN INTERACTING 9*‐like, Solyc08g059700), which has a conserved role in the control of organ size in both rosid and asterid species (Liu *et al*., 2020; Swinnen *et al*., 2022). We confirmed the differential expression of these four genes by qRT‐PCR in independently sampled IM/FM pools (Fig. 4g). Notably, checking the *tm3 stm3* DEG list of Zahn *et al*. (2023) revealed that the four genes are also derepressed in *tm3 stm3* mutant meristems (Fig. S10). We then also checked their expression in SYMs of WT, *mc*, and *ful2 mbp20* by qRT‐PCR and found them also derepressed there (Fig. S11). This indicates that these DEGs are more generally repressed by MADS‐domain transcription factors to promote reproductive fate. Their annotations suggest that this involves regulation of cell division, meristem maturation, and repression of undesired processes such as fatty acid biosynthesis or ABA signaling.

Several MADS‐box genes are upregulated in the *mc* and *ful2 mbp20* mutant meristems (Fig. S12). In particular, the upregulation of *MBP10* is interesting, given its proposed pseudogenization (Maheepala *et al*., 2019; Jiang *et al*., 2022). It is in line, however, with its upregulation in the *jointless* mutant reported by Huerga‐Fernández *et al*. (2024). The misexpression of related genes may interfere with the interpretation of the mutant phenotypes. For example, the upregulation of *MBP20*, *FUL1*, and *MBP10* in *mc* meristems could explain why some *mc* phenotypes are rather mild.

### Identification of putative *MC* or *FUL2/MBP20* unique DEGs

The expression data suggest that the stronger vegetative reversion phenotype in the *mc* mutant is a result of the more pronounced effect of MC on the target gene set shared with FUL2/MBP20. However, there appear to be a few *mc*‐specific DEGs that are differentially regulated in *mc* reproductive meristems (at least two‐fold) but expressed at WT levels in *ful2 mbp20* (Table S2; Dataset S3). This list is very short, with only 21 genes, and of these, we could only confirm the specific upregulation of *KAN2*‐like (*KANADI 2‐like*) and *SP* using qRT‐PCR on independently harvested FM‐IM samples (Figs 4h, S13). In particular, the specific upregulation of *SP* is interesting. While *SP* is stably repressed in the WT FM, its expression in the WT IM is highly variable (Table S2; Fig. 4h), possibly reflecting a transient role in the IM. In *mc* FMs, *SP* is strongly derepressed, with levels *c*. 20‐fold higher than the WT (Table S1). In *mc* IMs, *SP* levels are also higher, on average about threefold compared to WT IMs, but this is only significant in the FM‐IM qRT‐PCR data (Fig. S13).

We also identified 19 putative FUL2/MBP20‐specific genes (Table S2). However, specific expression could not be confirmed for selected DEGs in new FM‐IM samples or floral buds using qRT‐PCR (Figs S13, S14), with the exception of an orphan gene (Solyc12g062200), which was highly derepressed in *ful2 mbp20* FMs/IMs and in *mc ful2 mbp20* floral buds (Figs S13, S14; Table S2). In conclusion, MC appears to have a stronger effect on the acquisition of IM identity than FUL2/MBP20 because it is a more potent regulator of the common target gene set, but possibly also because it has a specific effect on *SP*. The stronger effect of MC on target gene expression may be caused by its higher expression level in IM/FM, but it is also possible that the AP1‐ and FUL‐like proteins bind with different affinities to the DNA of their target genes.

### Genome‐wide identification of the direct downstream targets of FUL2 and MC

To test whether the AP1‐ and FUL‐like TFs bind to the same genomic loci with equal affinity, or display different binding affinities, we performed DNA affinity purification sequencing (DAP‐seq). Because tomato AP1/FUL‐like proteins cannot form homodimers (Fig. 1b), they need to heterodimerize/tetramerize with other MADS‐domain proteins to bind CArG‐boxes and regulate target gene expression. Based on expression pattern (Fig. S15), interaction capacity, and function, we selected three interaction partners to perform the DAP‐seq assay with: J, J2, and TM3. These three TFs are involved in the different aspects of reproductive meristem development regulated by the AP1/FUL‐like proteins: TM3 promotes the floral transition (Alonge *et al*., 2020; Zahn *et al*., 2023), J2 acts in the FM to promote its maturation (Soyk *et al*., 2017), and J controls IM reproductive identity similar to MC (Quinet *et al*., 2006; Yuste‐Lisbona *et al*., 2016). We therefore performed DAP‐seq with FUL2 and MC in combination with J, J2, and TM3 to investigate whether FUL2 and MC are binding to different target gene sets. EMSA analysis with the different complexes revealed that they all form predominantly tetrameric complexes (Fig. S16). We identified 10 450, 4941, and 10 655 significantly enriched regions (peaks) for FUL2‐J, FUL2‐J2, and FUL2‐TM3, respectively, and 10 819, 4678, and 11 458 peaks for MC‐J, MC‐J2, and MC‐TM3 (*P* ≤ 0.0001) (Dataset S4).

**Fig. 5:**
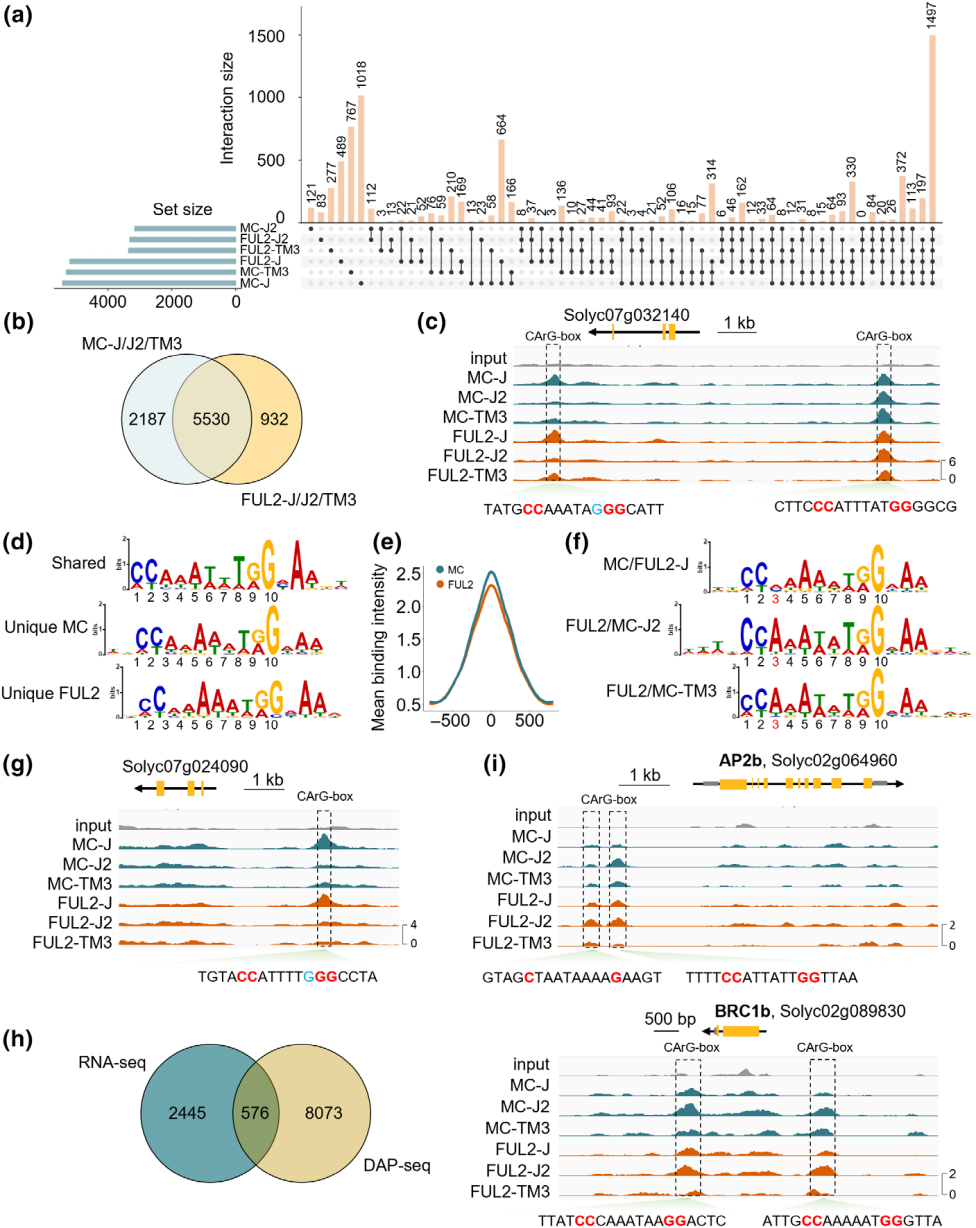
Genome‐wide overview of the downstream targets of FUL2 and MC in tomato. (a) UpSet plot showing the overlapping and unique target genes bound by protein complexes containing FUL2 or MC. The set size on the *x*‐axis defines the total number of bound genes. The *y*‐axis shows the number of genes in each category (connected dots). (b) Venn diagram showing the overlap of target genes of FUL2 and MC. (c) Integrative Genomics Viewer (IGV) screenshot visualizing the binding of protein complexes to a target gene for all six different complexes. Two CArG‐boxes are present at the locus, with the left one being a non‐canonical CArG‐box, which has a C in the A/T core. (d) PWM‐based models representing CArG‐box motifs obtained for FUL2 and MC based on (b). (e) Mean binding intensity of DAP‐seq peaks for FUL2 and MC. (f) PWM‐based models representing CArG‐box motifs obtained for J, J2, and TM3 based on (a). (g) IGV view of J binding with C variation at position 3 (or 8) of the CArG‐box motif, similar to the downstream CArG‐box in (c). (h) Overlap of differentially expressed genes of *mc/ ful2 mbp20* in the IM/FM, and genes at MC and FUL2 DAP‐seq peaks. (j) IGV screenshot of FUL2 and/or MC binding at *AP2b* and *BRC1b*.

We analyzed the genome‐wide distribution of the peaks for all protein complexes and found that *c*. 20–30% was in proximal regulatory regions (promotor ≤ 5 kb), while the vast majority were in distal intergenic regions (Fig. S17a). These numbers were different from an Arabidopsis DAP‐seq experiment that we recently performed for Arabidopsis FUL (Thoris *et al*., 2024), where only *c*. 4000 peaks were identified, of which > 75% were in the proximal regulatory region. However, the results are in line with other DAP‐seq experiments using larger genomes (e.g. potato, Shaikh *et al*., 2025). Probably, the large tomato genome offers many binding sites in (heterochromatic) intergenic regions that are not biologically relevant but still identified in *in vitro* approaches such as DAP‐seq. Therefore, the peaks of each protein complex were correlated with putative target genes by requiring the peaks to map within 10 kb upstream to 5 kb downstream of the gene. With this filtering, a variable number of potential direct targets was identified for each complex, with the MC‐J complex binding the most genes (5423) and MC‐J2 the least (3159) (Fig. 5a). By pooling the data of the three heterodimers, 6463 and 7718 genes were bound by FUL2 and MC, respectively, of which the majority (5530 genes) were bound by both proteins (Fig. 5b). Although this suggested that there are also unique binding sites for FUL2 and MC, inspection of the data in the IGV browser revealed that in cases where targets seemed specific, they were in fact just above the significance threshold for MC, and just below it for FUL2, or vice versa. We could not identify real FUL2‐specific or MC‐specific binding sites, as also illustrated by a scatter plot in which the coverage of the combined FUL2 targets was set out against that of the combined MC targets (Fig. S17b).

Because MADS‐domain proteins bind a highly conserved DNA sequence, called CArG‐box, as an obligate dimer, we were not surprised to observe that the CArG‐box was highly enriched under the peaks. We identified canonical CArG‐boxes (CC(A/T)_6_GG), underlying peaks bound with high affinity by the different complexes (Fig. 5c). No clear difference between PWMs of the pooled FUL2‐ and MC‐bound targets was identified (Fig. 5d). Additionally, we quantified the binding intensity of the common target regions based on normalized read coverage and found very similar values, although the MC peaks were slightly higher than those of FUL2 (Fig. 5e). Then, we extended the analysis to the different protein complexes and calculated the PWMs with pooled peaks for J, J2, and TM3. Interestingly, we found that the complexes with J have a relaxed constraint for the A/T stretch nucleotides, in particular at position 3 of the CArG‐box motif, where often a C is tolerated (Fig. 5f). Focusing on the MC‐J/FUL2‐J specific targets in the IGV browser, we indeed identified specific loci, or loci bound with higher affinity, which had C/G variation in the underlying motif (Figs 5c,g, S18a). This suggests that the MC‐J/FUL2‐J complexes exhibit a superior ability to bind genome‐wide targets compared to the J2 and TM3 complexes, which further explains that FUL2‐J and MC‐J have the largest number of bound target genes. Although FUL2 and MC have the same nucleotide binding preferences, the corresponding tetrameric complexes may exhibit a different affinity for CArG‐box spacing. For example, it has been found that the SEPALLATA (SEP) homotetramer does not exhibit spacing preferences, while the SEP‐AGAMOUS heterotetramer prefers a distinct spacing of ∼36, ∼47, or ∼57 bp between CArG box motifs (Lai *et al*., 2020). We tested whether we could identify spacing patterns for the six tested complexes but did not detect clear peaks (Fig. S17c). In conclusion, we identified differences in nucleotide binding affinity for the J‐containing complexes compared to the STM3 and J2 containing complexes, but there is no difference between the FUL‐ and AP1‐like proteins in their capacity to bind CArG‐boxes.

Next, we aimed to identify which differentially regulated genes described in the previous section are directly bound by FUL2/MC. Therefore, we compared the combined DEGs identified in *mc* and *ful2 mbp20* FMs/IMs at *P*adj < 0.05 with the MC/FUL2 DAP‐seq target genes from Fig. 5b and identified 576 potential direct targets of MC and FUL2/MBP20 in the FM/IM (Fig. 5h). Among these genes, the *AP2a*, *AP2b*, and *AP2c* genes are present, as well as, for example, *TM3, BRC1*, and *SQUAMOSA PROMOTER BINDING PROTEIN‐LIKE 15* (*SPL15*) (Figs 5i, S18b). However, other expected direct targets, such as *CKX5*, *CKX6*, and *CKX8*, did not show significant enrichment in the DAP‐seq, although we previously showed binding of FUL2 and MBP20 to their promoters using EMSA (Jiang *et al*., 2022). To test whether MC is also able to bind these *CKX* genes, we performed an EMSA experiment and confirmed binding with high affinity to *CKX5* and low affinity to *CKX6/8* (Fig. S19). In addition, no significant peaks were identified in the regulatory regions of the close homologs of *AHL15, WRI3, SlKIX9, WRKY28*, or *BLH1*, despite their strong upregulation in both the *mc* and *ful2 mbp20* mutants, nor was there significant enrichment at the *SP* locus. However, CArG‐boxes were identified in the promoter regions of most of these genes, including *SP* (Fig. S20), suggesting that they could be direct targets of MC/FUL2. This indicates that our DAP‐seq experiment has not picked up all relevant binding sites, possibly because the non‐amplified DAP library still contained DNA‐methylated sites.

### *SP* is a putative direct target of MC

**Fig. 6:**
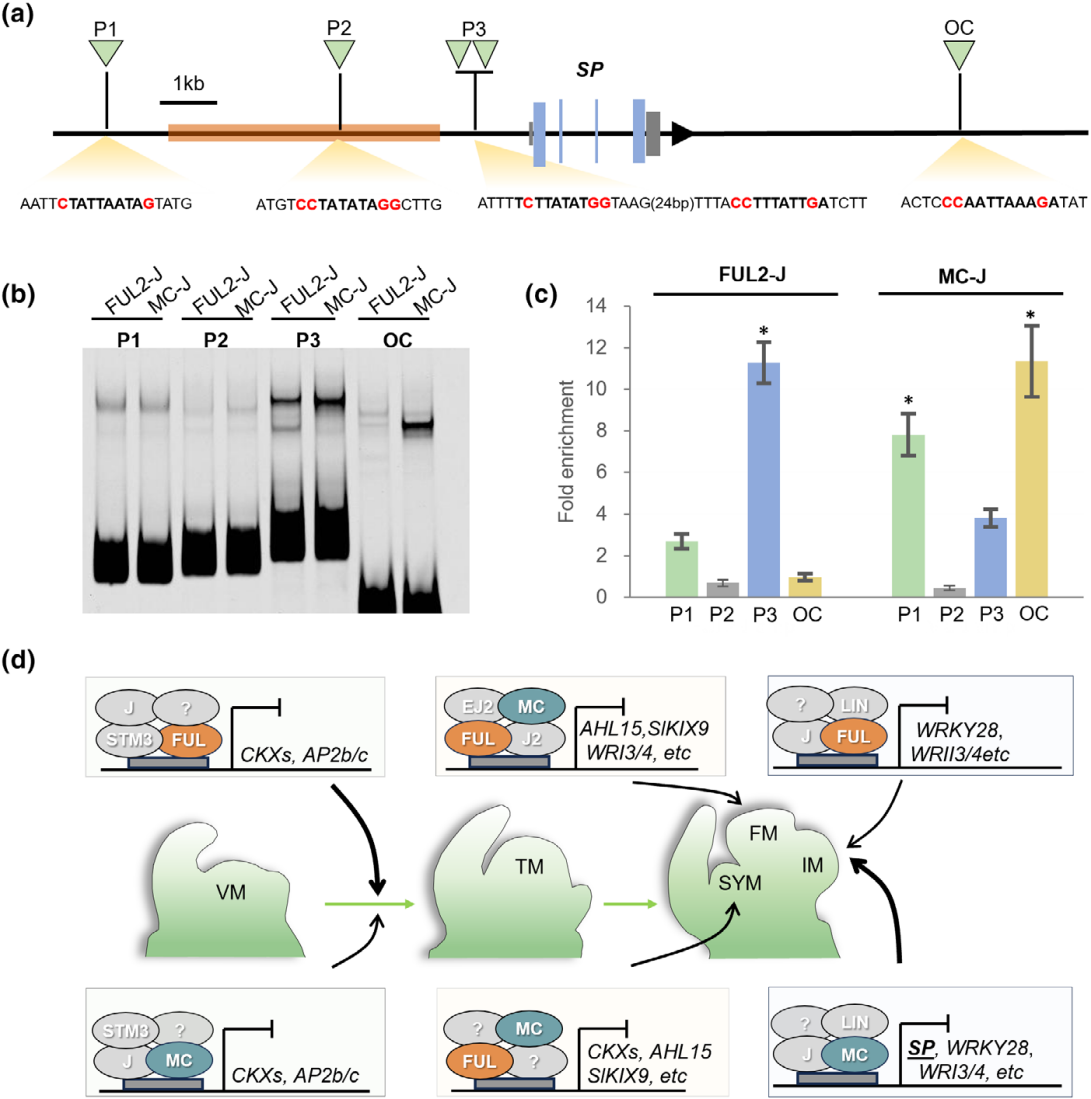
Targets of the MC/SlFUL complexes. (a) Schematic representation of the *SP* locus showing the location of CArG‐box motifs. P1–P3, tested probe fragments for EMSA; OC, EMSA probe fragment at open chromatin *c*. 5 kb downstream of the *SP* open reading frame. Orange bar, location of the retrotransposon. Blue bars, exons. (b) EMSA experiment testing binding of the MC‐J and FUL2‐J complexes to the probes depicted in (a). (c) DAP‐qPCR results using an amplified library and primers spanning the CArG‐boxes depicted in (a). Fold enrichment was calculated relative to the average value of two reference fragments without CArG‐box. Error bars indicate the SD based on three replicates. Significant differences were determined using a one‐way ANOVA with Tukey *post hoc* test (*, *P* < 0.001). (d) Model describing the putative functions of the AP1/FUL‐like TFs in tomato reproductive meristems. The model is based on data presented in this study, as well as data from other studies (see Discussion section), and built on a combination of expression data, protein–protein interaction data, and mutant analysis. Each complex is probably tetrameric, and putative interactors of FUL2/MC are indicated. Where the actions of FUL and MC are separated in different boxes, the width of the arrow that points towards the meristem indicates the contribution of MC in comparison to FUL. Dark gray rectangles indicate CArG‐box motifs. For connection to other key players, we refer to recently published reviews (e.g. Périlleux & Huerga‐Fernández, 2022).

Because we identified *SP* as the only specific target of MC that may explain its pronounced effect on IM identity, we investigated the *SP* locus in more detail (Fig. 6a). Despite the absence of significant DAP‐seq peaks, we did find several CArG‐boxes in the upstream region of *SP*. Since the Arabidopsis ortholog of MC, AP1, is binding in the 3' region of the *SP* ortholog *TFL1* (Kaufmann *et al*., 2010), we also inspected the downstream region and identified an additional CArG‐box (Fig. 6a). This CArG‐box is located in a small region with open chromatin (Fig. S21). Interestingly, we also identified a 4.7 kb retrotransposable element in the regulatory region of *SP*, *c*. 2 kb upstream of the start codon (Fig. 6a). This element, which has close homology to a Retrovirus‐related Pol polyprotein from transposon TNT 1–94, is not annotated at the *SP* locus in the ITAG4.0 genome version. It is present in *Solanum lycopersicum* and *S. pimpenellifolium*, but absent in *S. pennelli*, indicating a recent insertion. We tested using EMSA whether MC and FUL2 are able to bind to the identified CArG‐boxes, and found that a region *c*. 1.5 kb upstream of the ATG, with two non‐canonical CArG‐boxes separated by 32 bp (P3), was bound by both MC and FUL2 (Fig. 6b), while a CArG‐box further upstream (P2), lacking an A‐tract (Käppel *et al*., 2018), was not. Moreover, the downstream CArG‐box associated with open chromatin is strongly bound by MC alone (Fig. 6b). AP1 has been described as a pioneer transcription factor able to bind closed chromatin (Pajoro *et al*., 2014). Because *SP* is completely silenced before the primary floral transition, we reasoned that its locus may be repressed by epigenetic marks, and therefore difficult to access. We inspected the epigenetic marks at the *SP* locus and found that high levels of the repressive histone mark H3K27me3 occupy the entire *SP* locus up to the 3' open chromatin region (OC) (Fig. S21), while a high level of DNA methylation covers more specifically the upstream transposable element region, including the P3 CArG‐boxes just downstream of the element (Fig. S21d). We reasoned that the lack of DAP‐seq enrichment at the *SP* locus may be due to the use of a non‐amplified library from leaf tissue, which still contains DNA modifications (including DNA methylation) (O'Malley *et al*., 2016). Therefore, we amplified the library used for DAP‐seq and performed ampDAP‐qPCR with M‐J and FUL2‐J, using primers spanning the probe fragments. Interestingly, this showed enrichment for P1, P3, and OC but not for P2, in agreement with the EMSA results (Fig. 6b,c). Notably, the OC region was specifically highly enriched in the MC‐J sample, confirming the EMSA result, indicating that the tetrameric MC‐J complex can bind to the 3' region of *SP* similar to the situation in Arabidopsis, while FUL2‐J cannot.

Our results suggest that MC is required as an additional repressor to enforce *SP* silencing and maintain reproductive fate. In line with this, enhanced activity of *SP* in *35S:SP* lines causes the appearance of leaves in inflorescences, while *35S:SP* in the *an* mutant background (which only develops IMs) results in the complete conversion of these IMs to VMs (Pnueli *et al*., 1998). To find additional support for the importance of *SP* silencing during inflorescence development, we determined vegetative reversion rates in previously published *sp* mutants (Jiang *et al*., 2025) and found zero reversions, lower than the corresponding WTs (Fig. S6b). Thus, in the *mc* mutant, the *SP* locus may be more vulnerable for derepression, resulting in higher, albeit variable, *SP* expression and reversion to the vegetative phase.

## Discussion

### Dissecting the roles of *AP1/FUL‐like* genes during the tomato floral transition

Our data provide a detailed overview of the contributions of the *AP1*‐ and *FUL*‐like genes to tomato reproductive development, and show that together, the three genes are major controllers of both the floral transition and the acquisition/maintenance of reproductive fate in the IM, resulting in very few flowers in the *mc ful2 mbp20* triple mutant. We did not include *FUL1* and *MBP10* in our analysis, as the study of Jiang *et al*. (2022) revealed that the *ful2 mbp20* double mutant, and the *ful1 ful2 mbp10 mbp20* quadruple mutant (*quadful*) displayed very similar phenotypes, suggesting that *FUL1* and *MBP10* have at most a minor contribution. In line with this, our qPCR showed similar dysregulation of genes in *ful2 mbp20* and *quad‐ful* SYMs (Fig. 4b). However, given that mutation of *FUL1* mildly enhances the *ful2 mbp20* inflorescence phenotype (Jiang *et al*., 2022), knocking out *FUL1* in *mc ful2 mbp20* may reduce flower formation even more. Interestingly, while *MBP10* is not expressed in WT reproductive meristems and has been suggested to undergo pseudogenization (Maheepala *et al*., 2019; Jiang *et al*., 2022), its expression is derepressed in *mc* mutants (Fig. S12). This indicates, together with its upregulation in *j* mutants (Huerga‐Fernández *et al*., 2024), that *MBP10* may be able to compensate for the loss of related MADS‐box genes, similar to other MADS‐box genes, such as *STM3*, which were upregulated in the *ful2 mbp20* and *mc* mutants (Fig. S12). Thus, it is probable that knocking out all these genes would result in a complete block of flowering.

In the initiation of the floral transition, *FUL2* plays a prominent role due to its early expression (Jiang *et al*., 2022). Our data show that *MC* plays a minor role here, with a mild phenotype in the *mc* single mutant (Fig. 2b, Yuste‐Lisbona *et al*. (2016)), and a small contribution to the late‐flowering phenotype of the *ful2 mbp20* mutant in the triple mutant (Fig. 2b). The AP1/FUL TFs interact with the SOC1‐like proteins TM3/STM3 to form a flowering‐inducing complex, and this complex appears to regulate two main types of target genes directly: AP2‐like TFs (*AP2b/c* and *WRI3/4*) and cytokinin oxidases involved in the degradation of cytokinin (Jiang *et al*., 2022; Zahn *et al*., 2023) (see Fig. 6d for a hypothetical model of the mode‐of‐action of MC/FUL2/MBP20). In Arabidopsis, the *AP2*‐like TFs *AP2*, TARGET OF EARLY ACTIVATION TAGGED 1 (TOE1), TOE2, and TOE3, SCHLAFMUTZE (SMZ) and SCHNARCHZAPFEN (SNZ) are repressors of the floral transition through interaction with TOPLESS (TPL) co‐repressors (Aukerman & Sakai, 2003; Yant *et al*., 2009), and are direct targets of FUL (Balanzà *et al*., 2018). In addition, it was recently shown that the repression of *AP2* by SOC1 is needed to achieve meristem doming in Arabidopsis (Bertran Garcia de Olalla *et al*., 2024). Thus, it is likely that the tomato AP1/FUL TFs mainly control the floral transition together with the SOC1 co‐orthologs TM3/STM3 by repressing vegetative fate and allowing meristem doming through repression of *AP2*s and *CKXs* (Werner *et al*., 2003; Jiang *et al*., 2022; Wang *et al*., 2023) (Fig. 6d). J, the SVP ortholog interacting with the AP1/FUL/SOC1‐like proteins (Fig. S2), may contribute as well, given its abundance in VM and TM, and its effect on flowering time (Leseberg *et al*., 2008; Thouet *et al*., 2012; Jiang *et al*., 2022; Huerga‐Fernández *et al*., 2024). The *AP2*‐clade genes are probably additionally repressed via the age pathway, in which miR172 efficiently targets their transcripts (Wu *et al*., 2009; Lin *et al*., 2021). Because most species exhibit meristem doming upon the floral transition, it is plausible that the FUL/AP1/SOC1‐*AP2/CKX* module is a conserved mechanism in the onset of flowering.

Interestingly, we found that the crucial role of the tomato *AP1/FUL*‐like genes in the sympodial floral transition is not executed via the well‐known repressor *SP* (Pnueli *et al*., 1998; Thouet *et al*., 2008), or the *AP2*‐like genes, but appears primarily regulated via suppression of *CKXs* and other TFs, including homologs of *AHL15*, *WRI3/4*, *KIX9*, *WRKY28*, *and BLH1*. Possibly, this means that *AP2*‐like genes are already sufficiently repressed in the SYMs. Since *FUL2* expression was reported to be upregulated in an *sp* mutant background (Kang *et al*., 2022; Jiang *et al*., 2025), SP is probably acting upstream of *AP1/FUL‐like* genes in the SYM. Not surprising given its higher expression in SYMs, the comparison of the *ful2 mbp20* and *mc ful2 mbp20* phenotypes shows that *MC* has a much larger contribution to sympodial flowering time regulation than to primary shoot flowering. The specific effect that MC has on *SP* may also add to this function, although the upregulation of *SP* was not significant in *mc* SYMs (Fig. 4b).

### Dissecting the roles of *AP1/FUL*‐like genes in the establishment of FM and IM identity

In contrast to the reproductive transition, flower and inflorescence development are angiosperm‐specific processes. While most basal angiosperms have solitary flowers, more derived angiosperms have complex inflorescence structures that can be divided into different types, based on meristem determinacy, the presence/absence of a main axis, and occurrence of branching (Coen & Nugent, 1994). To evolve a more complex inflorescence, FM determination must be circumvented or bypassed. In tomato, where the latter occurs, reproductive meristem development requires a delicate balance between FM and IM identity, a trait that is in several species regulated by MADS‐domain complexes, sometimes in combination with the activity of the floral repressor TFL1 (Périlleux *et al*., 2019). Our transcriptomic data show that this balance has shifted in *mc* and *ful2 mbp20* mutants, but the careful separation of FM and IM meristems in WT, *mc*, and *ful2 mpb20* also reveals that the main function of the *AP1/FUL* genes in tomato appears to be the repression of vegetative identity, while we found no evidence that AP1/FUL directly regulates the *LFY‐UFO* module that controls FM identity and the upregulation of the floral organ identity genes in both the monocots and eudicots (Selva *et al*., 2021; Rieu *et al*., 2023a,b).

Tomato *AP1/FUL*‐genes are not essential for FM identity and patterning, so the first flower can form, albeit much later and with leaf‐like sepals (Fig. S5). FM maturation is delayed in *ful2 mbp20* mutants and in *mc ful2 mbp20* mutants, reflected in the inflorescence branching phenotype of both mutants, which form regularly additional IMs, like *j2 ej2* mutants (Soyk *et al*., 2017). MC and FUL2/MBP20 both appear to play a role in this process, but their exact contributions remain unclear because of the discrepancy between the phenotypic data (that show more branching in *ful2 mbp20* mutants) and the transcriptomic data (that show a more pronounced effect in the *mc* mutants). We suppose that their effect may depend on the environmental conditions, because inflorescence branching is a highly variable phenotype (Zahn *et al*., 2023). It may also depend on the extent to which they regulate *TM3/STM3*, which counteracts the branching effect (Wang *et al*., 2021, 2023; Zahn *et al*., 2023). Nevertheless, also in the *mc ful2 mbp20* triple mutant, the phenotype never reaches the severity of the *j2 ej2* branching phenotype. This suggests that J2 and EJ2 are more prominent regulators of FM maturation, acting also independently of *AP1/FUL‐like*.

It is *MC* that plays a major role in establishing reproductive fate in the IM, and we expected that the RNA‐seq comparison between *mc* IMs and *ful2 mbp20* FMs would shed light on the much more pronounced role of *MC*, but the transcript profiles were largely similar. A possible explanation for the more severe phenotype in the *mc* single mutant is simply that the higher dosage of *MC* in the wild‐type results in a more important functional contribution. However, the transcript abundances of *MBP20*, *FUL2*, and *FUL1* together almost add up to the *MC* levels, and expression level may therefore not be the only explanation. The role of MC is possibly enforced by its specific effect on the repression of *SP*. In Arabidopsis, the mutual regulation between *TFL1* and *AP1* is essential to gain the strict boundaries between the indeterminate IM and the FMs that form on its flanks (Liljegren *et al*., 1999; Kaufmann *et al*., 2010; Goslin *et al*., 2017; Zhu *et al*., 2020). TFL1 is directly suppressing both *AP1* and *LFY*, thereby retaining reproductive identity, but preventing floral organ initiation (Zhu *et al*., 2020), while AP1 is on its turn directly suppressing *TFL1* (Kaufmann *et al*., 2010; Goslin *et al*., 2017). The upregulation of *SP* in the *mc* mutant may point in the same direction. In tomato, MC may be required to keep *SP* completely repressed and sustain reproductive identity (Fig. 6). It may be able to fulfill this role, in contrast to FUL2/MBP20, because of its higher ability to access closed chromatin (Pajoro *et al*., 2014), and/or because it is better capable to bind the 3' CArG‐box. Possibly, the MC‐J tetrameric complex connects the 3' CArG‐box with one of the upstream CArG‐boxes (P1 or P3) to establish a repressive loop. The fact that mutations in the MC interactor J also lead to *SP* upregulation (Huerga‐Fernández *et al*., 2024) do support a role for MC‐J in the repression of *SP* to maintain reproductive fate in the IM. Also, the IM‐marker LIN, which physically interacts with MC, may act together with MC (and J) in a complex (Fig. 6d). It is not yet clear why AP1‐like TFs would have the capacity to repress *TFL1*‐like genes and FUL‐like TFs would not, but we find here that the 3' CArG‐box may be key to the answer. Another possibility is that, because AP1 can interact with more non‐MADS proteins than other ABC‐type MADS‐domain TFs (Smaczniak *et al*., 2012), including the co‐repressors LEUNIG and ZEUSS, it may exhibit additional functionality. This functionality may be acquired via its specific C‐terminal motif, which arose through a frameshift after the split of the AP1‐ and FUL‐clades (Litt & Irish, 2003). In addition to the repression of *SP*, the repression of *AP2b*, *AP2c*, *AHL15*, *WRI3*, *KIX9*, *WRKY28*, and *BLH1* by the joint activity of MC and SlFULs further ensures reproductive fate and meristem maturation (Schmitz *et al*., 2005; Pagnussat *et al*., 2007; To *et al*., 2012; Ding *et al*., 2013; Kim *et al*., 2013; Zhao *et al*., 2018; Karami *et al*., 2020; Tian *et al*., 2020; Swinnen *et al*., 2022).

It is clear that for the formation of the Arabidopsis inflorescence, the AP1/LFY‐TFL1 module is very important to separate IM and FM fate and to ensure continuous IM growth, while *FUL* plays a minor role in this process (Ferrándiz *et al*., 2000). In tomato, on the other hand, the *AP1/FUL* genes are only modestly involved in the regulation of FM maturation but are crucial for the maintenance/gain of reproductive fate in the freshly formed IMs. Thus, the role of the *AP1/FUL* genes in FM identity and patterning appears less important in tomato than in Arabidopsis. While flower formation is abolished in the Arabidopsis *ap1 cal* double mutant (Ferrándiz *et al*., 2000), the first flower in tomato *mc* or *mc ful2 mbp20* mutants is normal, except for leaf‐like sepals. In tomato, the main function of the *AP1/FUL* genes appears to be the control of reproductive meristem fate, while the gradual transition of IM to FM and subsequent initiation of the floral organs is mainly regulated via J2 EJ2 and the S‐FA‐AN module. Thus, both in Arabidopsis and tomato, the FT/SPL‐AP1/FUL‐*CKX/AP2* module regulates the floral transition and reproductive identity, while the FT‐LFY‐UFO‐ABC‐class module regulates FM identity, patterning, and floral organ initiation. Because *FUL*‐like genes in monocots are essential for the reproductive transition and inflorescence architecture as well (e.g. Preston & Kellogg, 2007; Li *et al*., 2019), the role of the FUL/AP1‐like genes in regulating these traits together appears very conserved. After the split of the euAP1, euFULI, and euFULII clades in the eudicots, divergence resulted in a gain of function for the euFULI‐clade genes in fruit development (Pabón‐Mora *et al*., 2012), while the AP1 lineage became important for sepal identity. Furthermore, our data suggest that AP1‐like TFs have a conserved role in the regulation of *TFL1* orthologs. In conclusion, we do show here that genes from all three clades contribute to reproductive meristem identity and development in the eudicots but that there are lineage‐specific differences in the contributions of the *FUL*‐ and *AP1*‐like genes. These are mainly linked to their expression patterns, but AP1 may also have additional functionality on the protein level.

## Competing interests

None declared.

## Author contributions

MB conceived the project; XJ, IEZ, KT, ER and CR performed the research; MB, XJ, IZ and KT analyzed the data and prepared the figures; GCA, CG‐M. CF and MB supervised the project. MB and HW acquired project funding. MB, XJ and IEZ wrote the article with input from GCA and KT; all authors read and approved the publication of the manuscript.

## Disclaimer

The New Phytologist Foundation remains neutral with regard to jurisdictional claims in maps and in any institutional affiliations.

## Supporting information


**Dataset S1** Marker genes in FM and IM.

**Dataset S2** Venn analysis of DEGs in Fig. 4(e).

**Dataset S3** RNA‐seq data overview and DEG lists.

**Dataset S4** List of DAP‐Seq peaks bound by FUL2‐ and MC‐ complexes.

**Fig S1** The protein sequences of tomato AP1/FUL‐like proteins.
**Fig. S2** Yeast two‐hybrid analysis of tomato AP1/FUL‐like proteins with MADS‐domain proteins from different subfamilies.
**Fig. S3** Localization of *FUL1, FUL2*, *MBP20*, and *MC* transcripts in WT reproductive meristems by *in situ* hybridization using specific 5′/3′ probes.
**Fig. S4** Quantification of flowering time of WT and *mc ful2 mbp20* mutants under glasshouse conditions in autumn.
**Fig. S5** Flower and inflorescence phenotypes of WT and *ap1/ful*‐like mutants.
**Fig. S6** Inflorescence vegetative reversion in *ful1 ful2 mbp20* mutants.
**Fig. S7** Target genes in the sympodial shoot vegetative meristem (SVM) tested with qRT‐PCR in different genotypes.
**Fig. S8** Expression dynamics of marker genes during reproductive meristem development.
**Fig. S9** PCA plot of the RNA‐Seq samples.
**Fig. S10** FPKM values of selected DEGs across vegetative and reproductive meristem stages in WT and *tm3 stm3* mutant.
**Fig. S11** Gene expression detected in SYMs of WT, *mc*, and *ful2 mbp20* mutants by qRT‐PCR.
**Fig. S12** Z‐normalized expression of MADS‐box genes in the sFM and sIM of WT, *mc*, and *ful2 mbp20*.
**Fig. S13** Expression analysis of DEGs from the RNA‐seq experiment in mixed FM/IM using qRT‐PCR.
**Fig. S14** Expression of putative MC‐specific and FUL2/MBP20‐specific DEGs tested by qRT‐PCR in young floral buds of WT, *mc*, *ful2 mbp20, quad‐ful*, *and mc ful2 mbp20*.
**Fig. S15** Expression of *J*, *J2*, and *TM3/STM3* in shoot apical meristems.
**Fig. S16** Electrophoretic Mobility Shift Assay (EMSA) to test complex formation of the different combinations of MADS‐domain proteins.
**Fig. S17** Analysis of DAP‐seq peaks.
**Fig. S18** Integrative Genomics Viewer (IGV) screenshots of targets with clear peaks.
**Fig. S19** MC can bind to the promoters of *CKX5/6/8*.
**Fig. S20** Integrative Genomics Viewer (IGV) screenshots of DEGs with no significant DAP‐seq peaks.
**Fig. S21** DNA and chromatin marks up‐nd downstream of *SP*.
**Table S1** Primers used in this study.
**Table S2** FPKM values of interesting DEGs.
**Table S3** Tomato gene accession numbers.
Please note: Wiley is not responsible for the content or functionality of any Supporting Information supplied by the authors. Any queries (other than missing material) should be directed to the *New Phytologist* Central Office.

## Data Availability

The raw RNA‐Seq data are publicly available in the Sequence Read Archive (SRA, ncbi.nlm.nih.gov/sra) with the accession number PRJNA1006186. The raw DAP‐Seq data are publicly available in the Gene Expression Omnibus (GEO, https://www.ncbi.nlm.nih.gov/geo/) with the accession number GSE271397. Gene sequences can be found in the Sol Genomics Network database (http://solgenomics.net/) under the accession numbers in Table S3.
