# Supplementary material for: Tomato flowering depends on overlapping functions of AP1/FUL‐like genes in reproductive meristem specification
Source: New Phytol. 2025 Aug 19;248(2):1002–20. doi: 10.1111/nph.70451 (PMC12445816; doi:10.1111/nph.70451)
Supplement: Supplementary file 5 — Fig S1 The protein sequences of tomato AP1/FUL‐like proteins. Fig. S2 Yeast two‐hybrid analysis of tomato AP1/FUL‐like proteins with MADS‐domain proteins from different subfamilies. Fig. S3 Localization of FUL1, FUL2, MBP20, and MC transcripts in WT reproductive meristems by in situ hybridization using specific 5′/3′ probes. Fig. S4 Quantification of flowering time of WT and mc ful2 mbp20 mutants under glasshouse conditions in autumn. Fig. S5 Flower and inflorescence phenotypes of WT and ap1/ful‐like mutants. Fig. S6 Inflorescence vegetative reversion in ful1 ful2 mbp20 mutants. Fig. S7 Target genes in the sympodial shoot vegetative meristem (SVM) tested with qRT‐PCR in different genotypes. Fig. S8 Expression dynamics of marker genes during reproductive meristem development. Fig. S9 PCA plot of the RNA‐Seq samples. Fig. S10 FPKM values of selected DEGs across vegetative and reproductive meristem stages in WT and tm3 stm3 mutant. Fig. S11 Gene expression detected in SYMs of WT, mc, and ful2 mbp20 mutants by qRT‐PCR. Fig. S12 Z‐normalized expression of MADS‐box genes in the sFM and sIM of WT, mc, and ful2 mbp20. Fig. S13 Expression analysis of DEGs from the RNA‐seq experiment in mixed FM/IM using qRT‐PCR. Fig. S14 Expression of putative MC‐specific and FUL2/MBP20‐specific DEGs tested by qRT‐PCR in young floral buds of WT, mc, ful2 mbp20, quad‐ful, and mc ful2 mbp20. Fig. S15 Expression of J, J2, and TM3/STM3 in shoot apical meristems. Fig. S16 Electrophoretic Mobility Shift Assay (EMSA) to test complex formation of the different combinations of MADS‐domain proteins. Fig. S17 Analysis of DAP‐seq peaks. Fig. S18 Integrative Genomics Viewer (IGV) screenshots of targets with clear peaks. Fig. S19 MC can bind to the promoters of CKX5/6/8. Fig. S20 Integrative Genomics Viewer (IGV) screenshots of DEGs with no significant DAP‐seq peaks. Fig. S21 DNA and chromatin marks up‐nd downstream of SP. Table S1 Primers used in this study. Table S2 FPKM values of interesting DEGs. Tabl [file NPH-248-1002-s002.pdf]

New Phytologist supporting information

Article title: Tomato flowering depends on overlapping functions of *AP1/FUL*-like genes in reproductive meristem specification.

Authors: Xiaobing Jiang, Iris E. Zahn, Kai Thoris, Chris Roelofsen, Edelin Roque, Concepción Gómez-Mena, Cristina Ferrándiz, Hongru Wang, Gerco C. Angenent and Marian Bemer

Article acceptance date: 18 July 2025

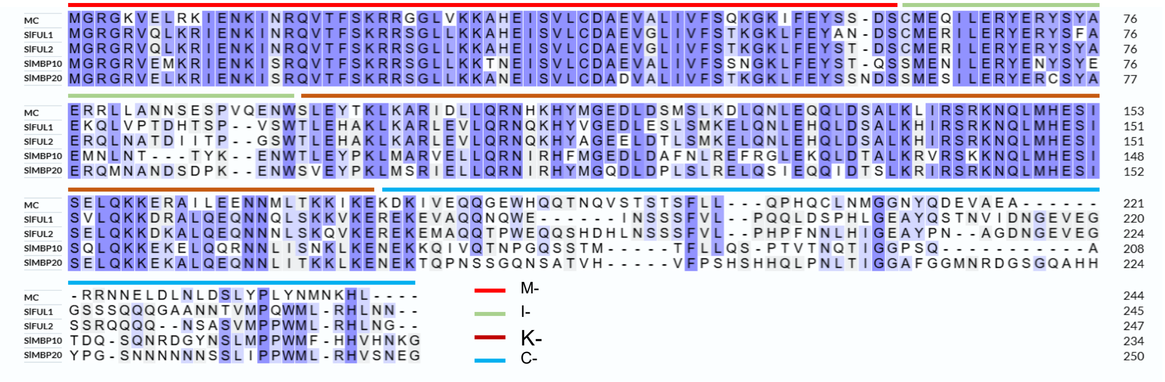

Fig S1. The protein sequences of tomato AP1/FUL-like proteins. The entire M-, I-, K-domains, and divergent C-domains are displayed.

Subfamily	Protein	MC	FUL1	FUL2	MBP10	MBP20
SVP	SlMBP24	-	-	-	-	-
	J	+	+	+	+	+
AG	TAG1	-	-	+	-	-
SEP	TM5	+	-	+	-	+
	TM29	-	-	+	-	-
	MADS-RIN	+	+	+	-	+
	MADS1/EJ2	+	+	+	-	-
	SlMBP21/J2	+	+	+	+	+
SOC1	TM3	+	+	+	+	+
	STM3	+	+	+	+	+
	SlMBP18	+	+	+	-	-
	SlMBP13	-	-	-	-	-
	SlMBP14	-	-	-	-	-
ANR1	SlMBP9	-	-	-	-	-
	SlMBP12	-	-	-	-	-
TT16	SlMBP22	-	-	-	-	-
AP1/FUL	MC	-	-	-	-	-
	FUL1	-	-	-	-	-
	FUL2	-	-	-	-	-
	MBP10	-	-	-	-	-
	MBP20	-	-	-	-	-

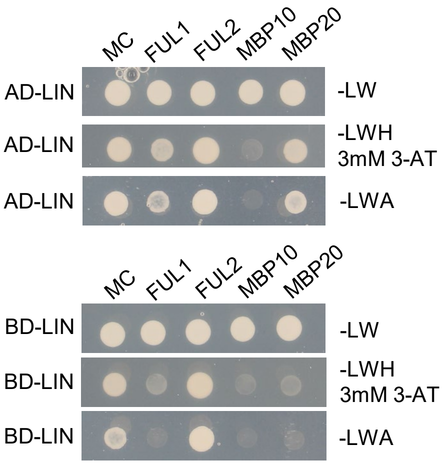

Fig. S2. Yeast two-hybrid analysis of tomato AP1/FUL-like proteins with MADS-domain proteins from different subfamilies. Reciprocal interaction is shown by dark yellow shading, one-way interaction by light yellow shading, and blank cells indicate a failure to interact. Data combined from Jiang et al, 2022, and this study. The results for LIN are new for FUL1, FUL2, MBP10 and MBP20 and therefore presented in a separate panel.

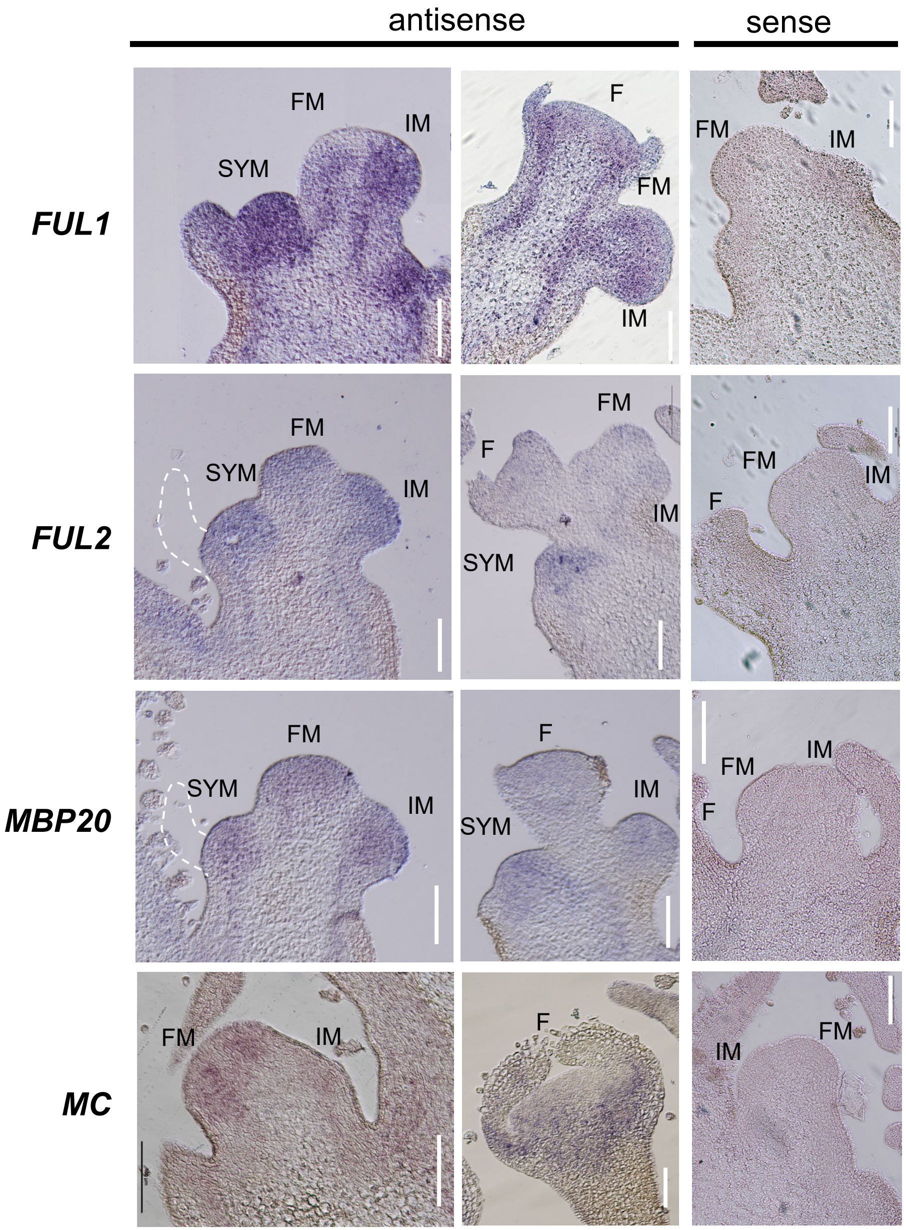

Fig S3. Localization of *FUL1, FUL2*, *MBP20* and *MC* transcripts in WT reproductive meristems by in situ hybridization using specific 5'/3' probes. Left two panels: antisense probe; right panel: sense probe. FM: floral meristem, IM: inflorescence meristem, SYM, sympodial shoot meristem, F: flower. White bar: 100 μm.

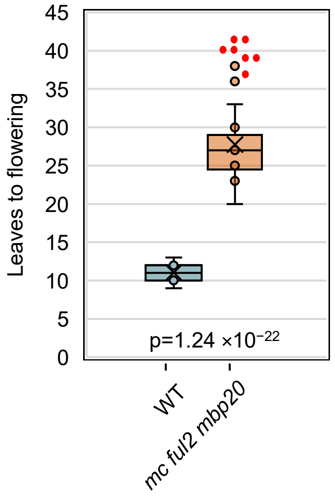

Fig. S4. Quantification of flowering time of WT and *mc ful2 mbp20* mutants under greenhouse conditions in autumn. The dots represent individual plants (WT n=21, mc ful2 mbp20 n=37). The red dots show the number of formed leaves for individual *mc ful2 mbp20* mutants that had not yet made the floral transition when the experiment ended (n=7 (out of 37)). The boxplots show the flowering time before the floral transition, excluding the red data points. The p-value was calculated using a two-tailed independent Student’s t-test.

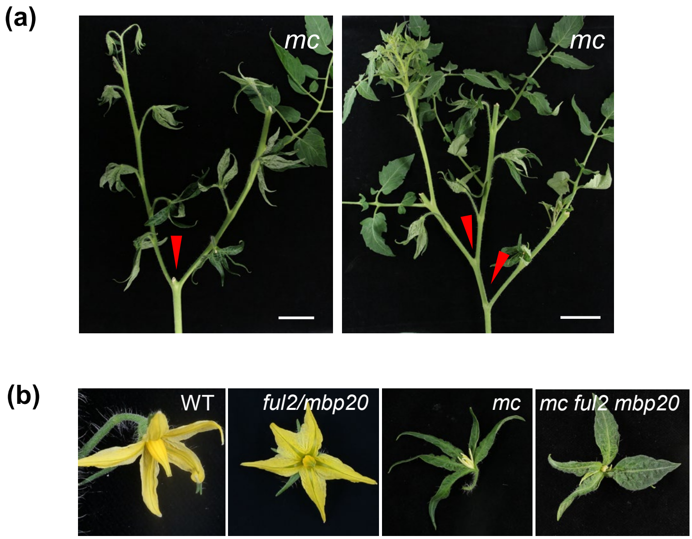

Fig. S5. Flower and inflorescence phenotypes of WT and *ap1/ful*-like mutants. (a) Branched inflorescences in the *mc* mutant. Red arrows indicate branching points. White bar: 1cm. (b) Flowers of WT and the three different mutants.

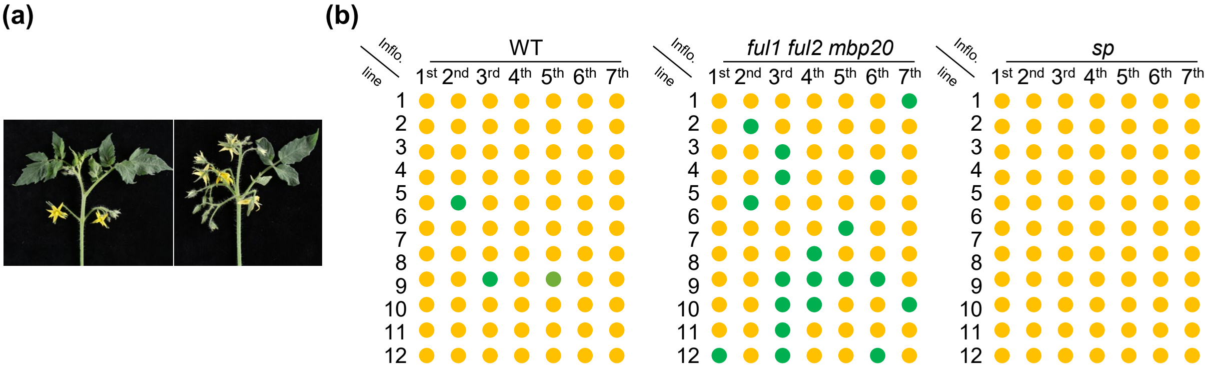

Fig. S6. Inflorescence vegetative reversion in *ful1 ful2 mbp20* mutants. (a) Representative inflorescences that reverted to shoot growth (left panel) and leaf development (right panel) in a *ful1 ful2 mbp20* mutant. (b) Frequency of inflorescence vegetative reversion in WT and *ful1 ful2 mbp20* mutant. Dots in yellow indicate normal inflorescences and green indicate vegetative reverted inflorescences.

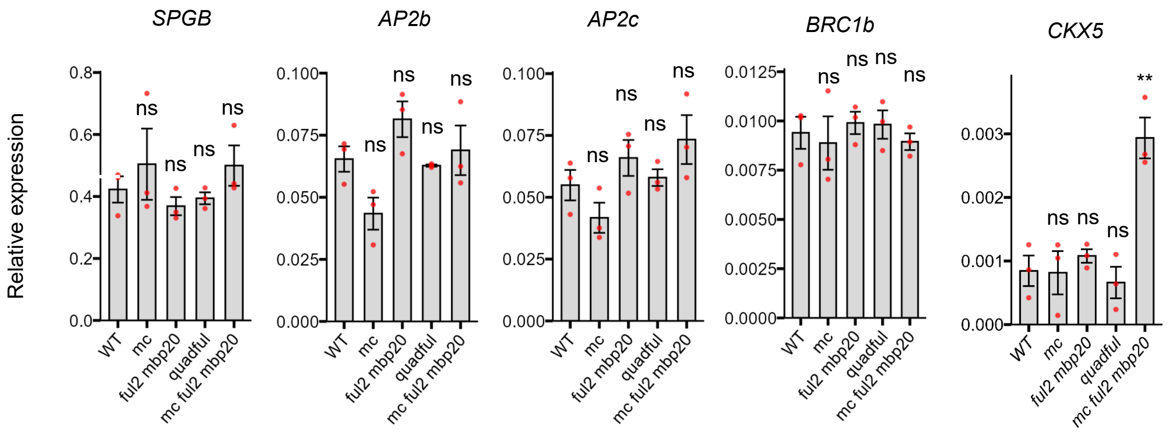

Fig. S7. Target genes in the sympodial shoot vegetative meristem (SVM) tested with qRT-PCR in different genotypes. Values represent the mean ± SE of three biological replicates (indicated with red dots). Significant differences were determined using a two-tailed Student's t-test (* $p < 0.05$, ** $p < 0.01$). ns: non-significant.

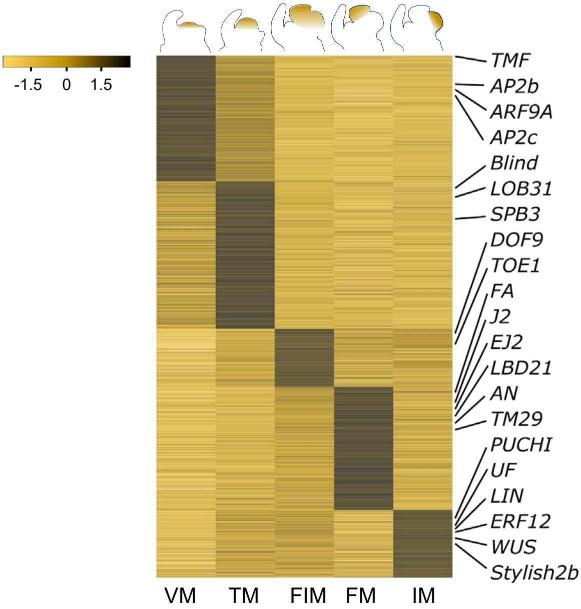

Fig. S8. Expression dynamics of marker genes during reproductive meristem development. Z-normalized expression of meristem-maturation markers in the primary VM, TM, FM/IM, and sFM, sIM. Genes were clustered based on developmental stage with the highest expression level. Schematic apices show isolated meristems highlighted in yellow-brown. Scaled expression was calculated based on the average expression values from three (VM, TM, FM/IM) or four biological replicates (sFM, sIM).

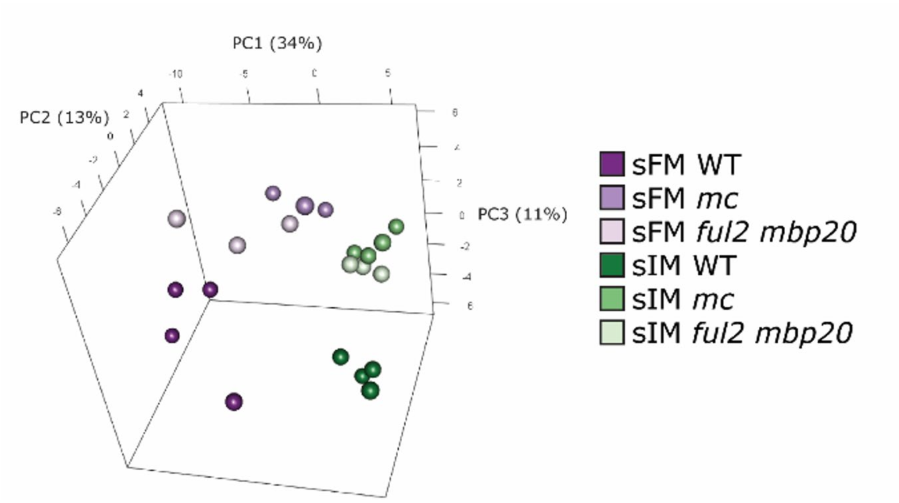

Fig. S9. PCA plot of the RNA-Seq samples. Note that PC1 represents the developmental stage of the meristems, as negative PC1 values correlate with sFM identity and positive PC1 values with sIM identity.

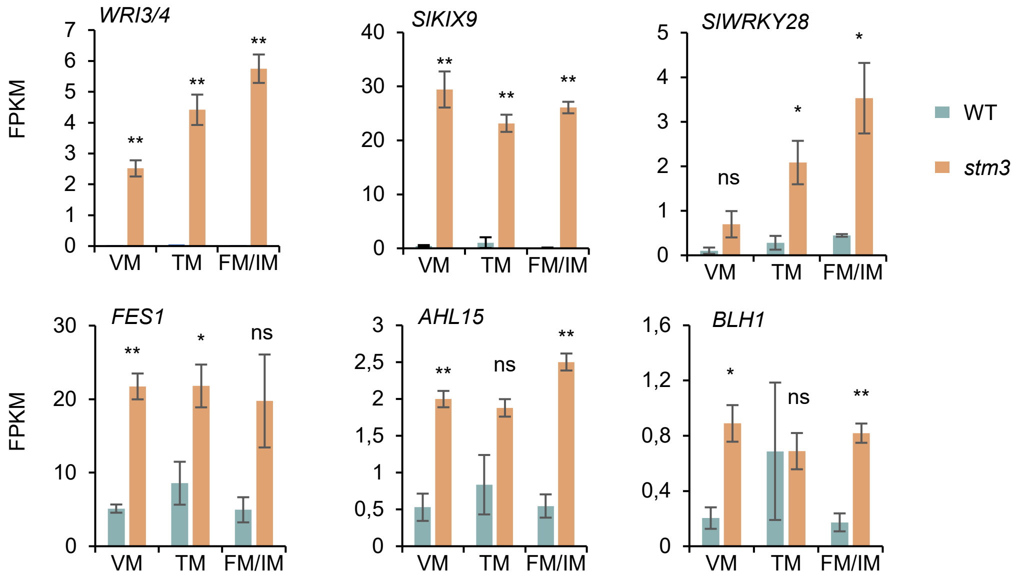

Fig. S10. FPKM values of selected DEGs across vegetative and reproductive meristem stages in WT and *tm3 stm3* mutant (from Zahn et al., 2023)**.** The values shown (mean ± SE) are the average of three replicates. Significant differences were calculated using a two-tailed Student's *t* test (* $p < 0.05$ and ** $p < 0.01$). VM: vegetative merisem, TM: transition meristem, FM/IM: floral meristem and inflorescence meristem.

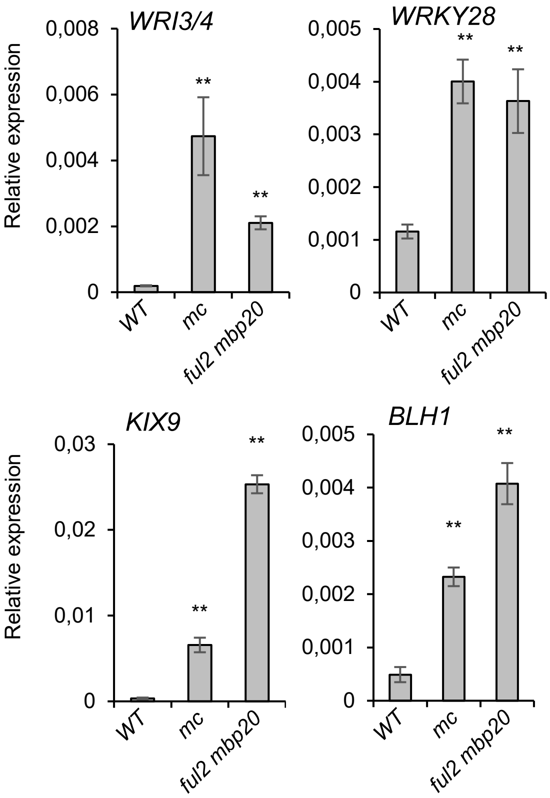

Fig. S11. Gene expression detected in SYMs of WT, *mc*, and *ful2 mbp20* mutants by qRT-PCR. The values shown (mean ± SE) are the average of three replicates. Significant differences were calculated using a two-tailed Student's *t* test (* $p < 0.05$ and ** $p < 0.01$).

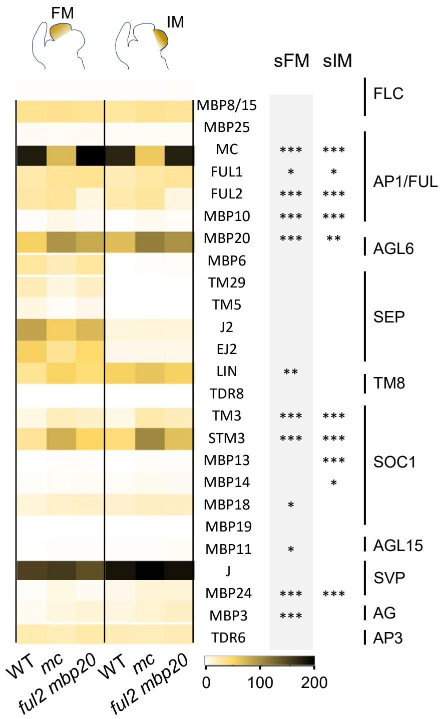

Fig. S12. Z-normalized expression of MADS-box genes in the sFM and sIM of WT, *mc* and *ful2 mbp20*. Asterisks indicate statistical significance according to the adjusted p value from the likelihood-ratio test (LRT) between WT, *mc* and *ful2 mbp20* in the sFM or sIM. All MADS-MIKC type genes with substantial expression (FPKM>1) in at least one sample type are shown. The non-normalized expression of these and the 15 more lowly expressed genes are shown in Supplementary Data Set 4. Schematic apices show isolated meristems highlighted in yellow-brown. MADS-box gene subfamilies are based on Smaczniak et al. (2012) and Wang et al. (2019). * Padj < 0.05, ** Padj < 0.01, *** Padj < 0.001.

Common targets

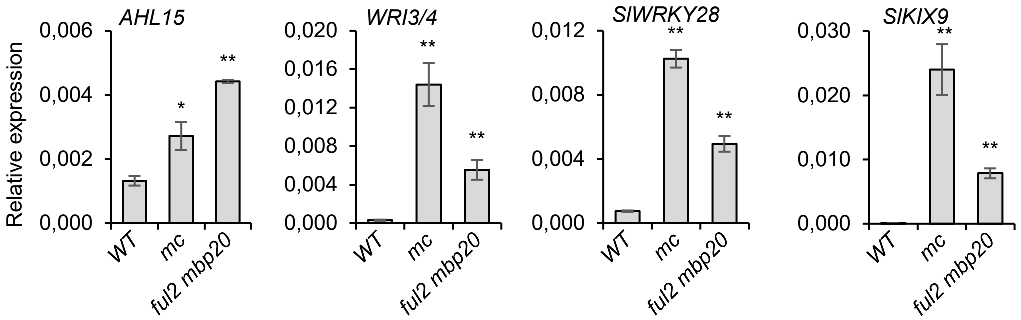

MC Specific targets

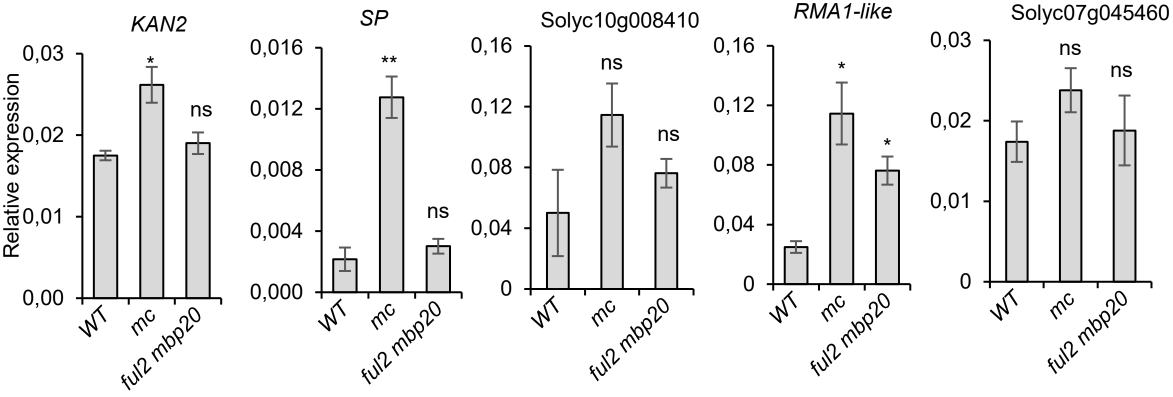

FUL2 Specific targets

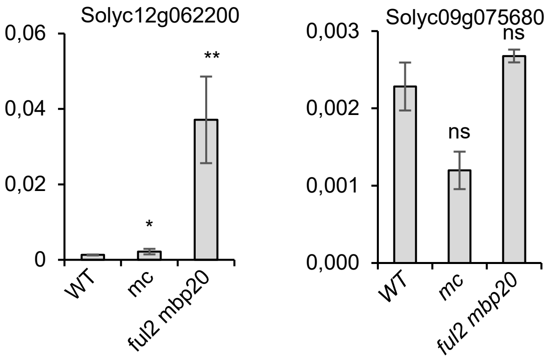

Fig. S13. Expression analysis of DEGs from the RNA-seq experiment in mixed FM/IM using qRT-PCR. The values shown (mean ± SE) are the average of three replicates. Significant differences were calculated using a two-tailed Student's *t* test (* $p < 0.05$ and ** $p < 0.01$).

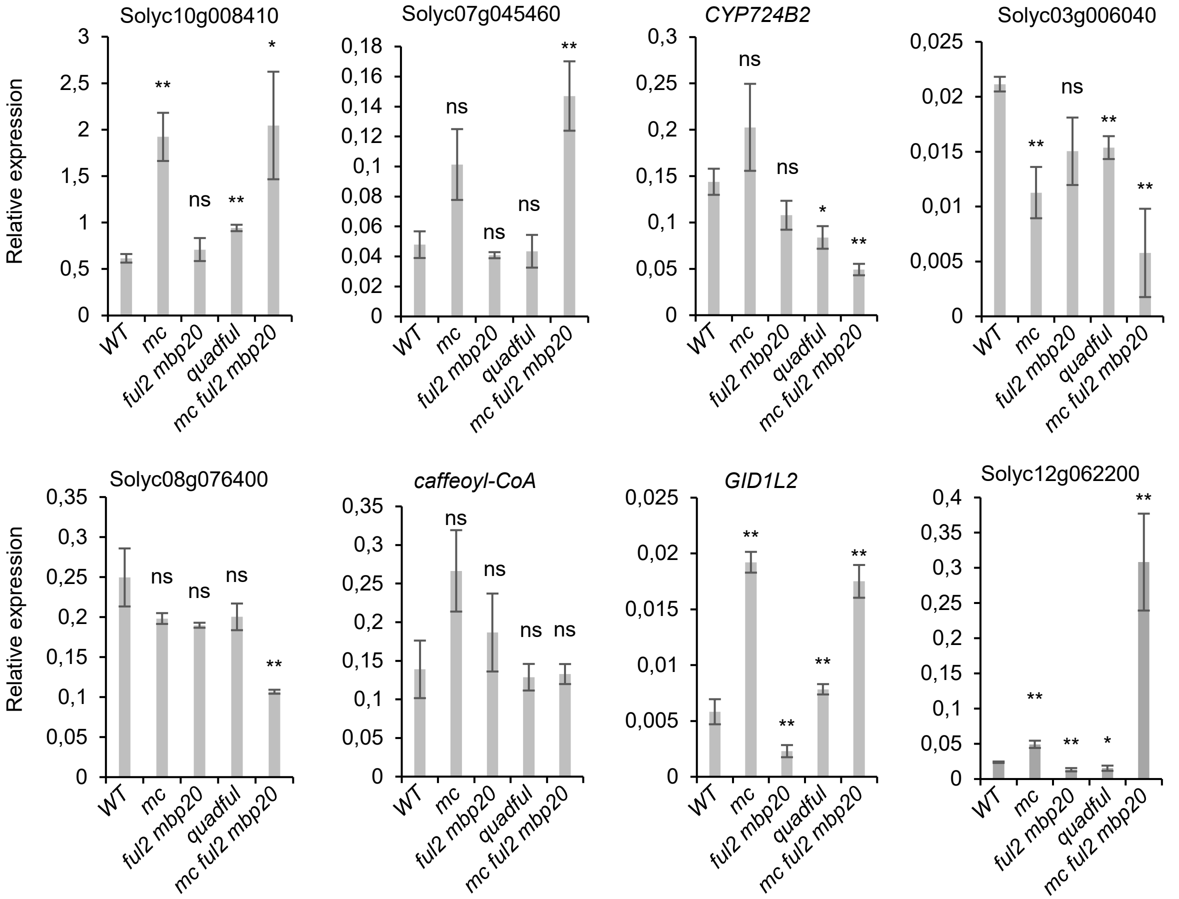

Fig. S14. Expression of putative MC-specific, and FUL2/MBP20-specific DEGs tested by qRT-PCR in young floral buds of WT, *mc*, *ful2 mbp20, quad-ful and mc ful2 mbp20*. The values shown (mean ± SE) are the average of three replicates. Significant differences were calculated using a two-tailed Student's *t* test (* $p < 0.05$ and ** $p < 0.01$).

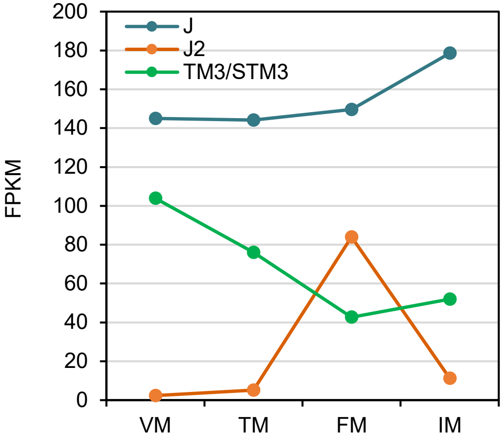

Fig. S15. Expression of *J*, *J2*, and *TM3/STM3* in shoot apical meristems. VM: vegetative meristem; TM: transition meristem; FM: floral meristem; IM: inflorescence meristem.

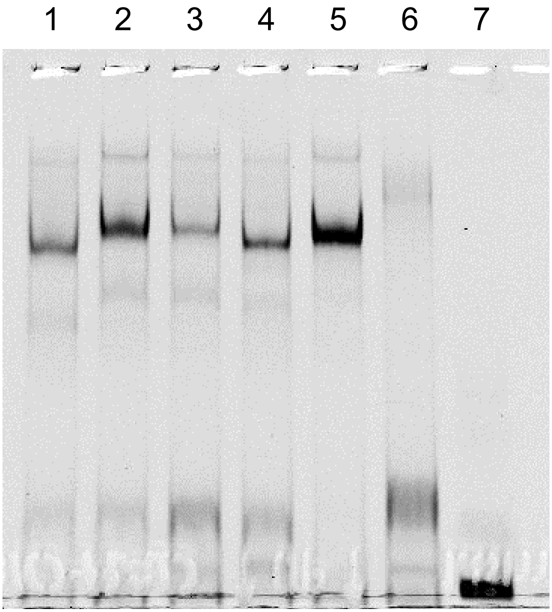

	Protein	Probe
**1.**	J2	SAUR10
**2.**	FUL2 + J2	SAUR10
**3.**	MC + J2	SAUR10
**4.**	MC + TM3	SAUR10
**5.**	FUL2 + TM3	SAUR10
**6.**	TNT -	SAUR10
**7.**	MQ -	SAUR10

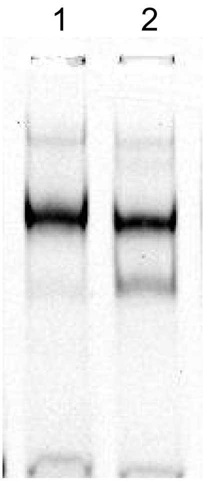

	Protein	Probe
**1.**	MC+J	SAUR10
**2.**	FUL2+J	SAUR10

Fig. S16. Electrophoretic Mobility Shift Assay (EMSA) to test complex formation of the different combinations of MADS-domain proteins. Combinations of two proteins were added to the CArG-box containing SAUR10 probe (Bemer et al., 2017). The FUL2 and MC proteins were fused to a FLAG-tag. Tetrameric complexes are formed in all cases. Only in the case of FUL2-J, a prominent dimeric complex is also visible.

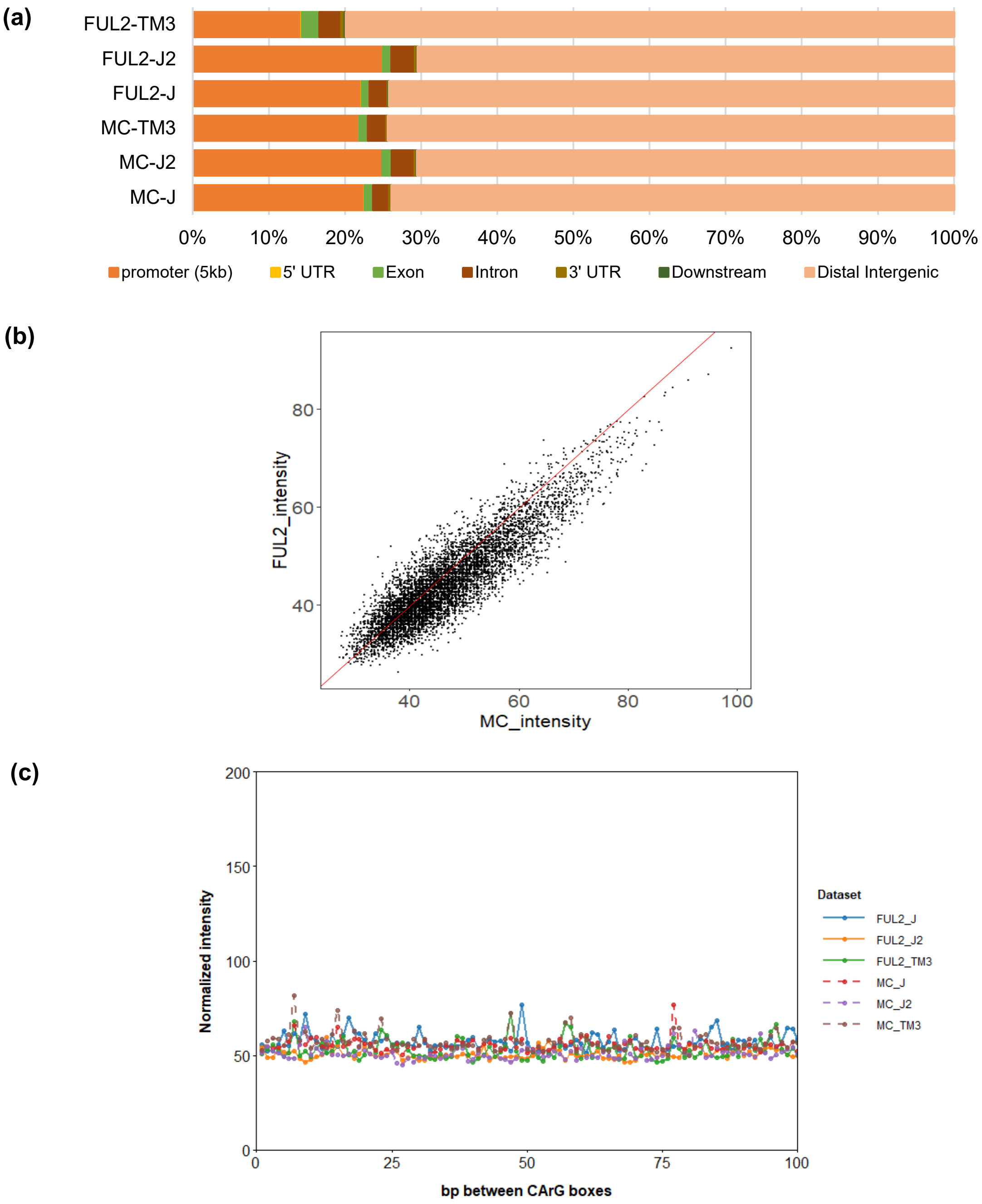

Fig. S17. Analysis of DAP-seq peaks. (a) distribution across gene features. (b) comparison of peak intensities for the pooled FUL2 and MC common targets at $p<0.0001$. (c) Normalized intensity of all peak pairs with given CArG-box spacing.

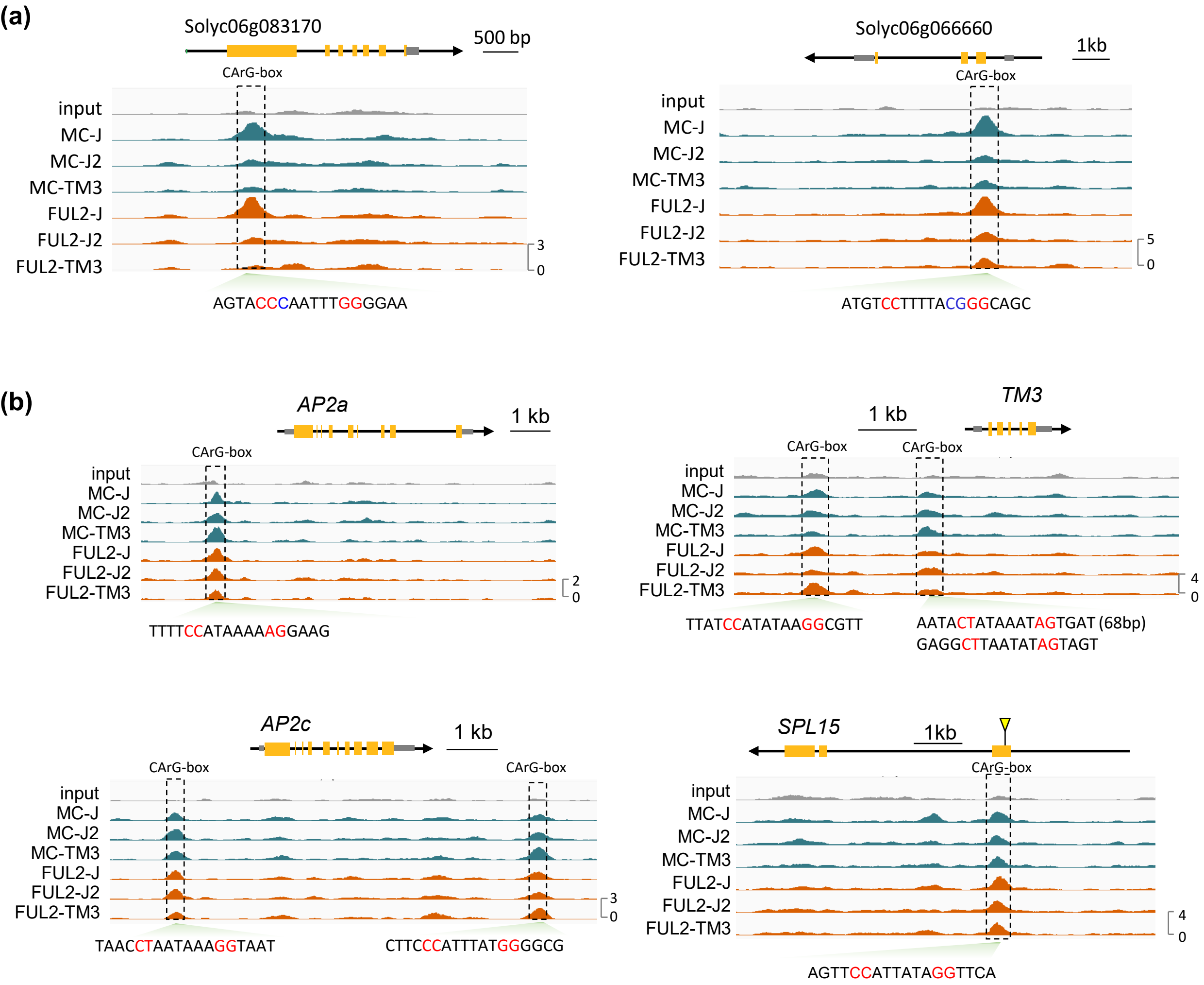

Fig. S18. Integrative Genomics Viewer (IGV) screenshots of targets with clear peaks. (a) J binding with C variation at position 3 of CArG-box motif. (b) DEGs bound by FUL2 and MC.

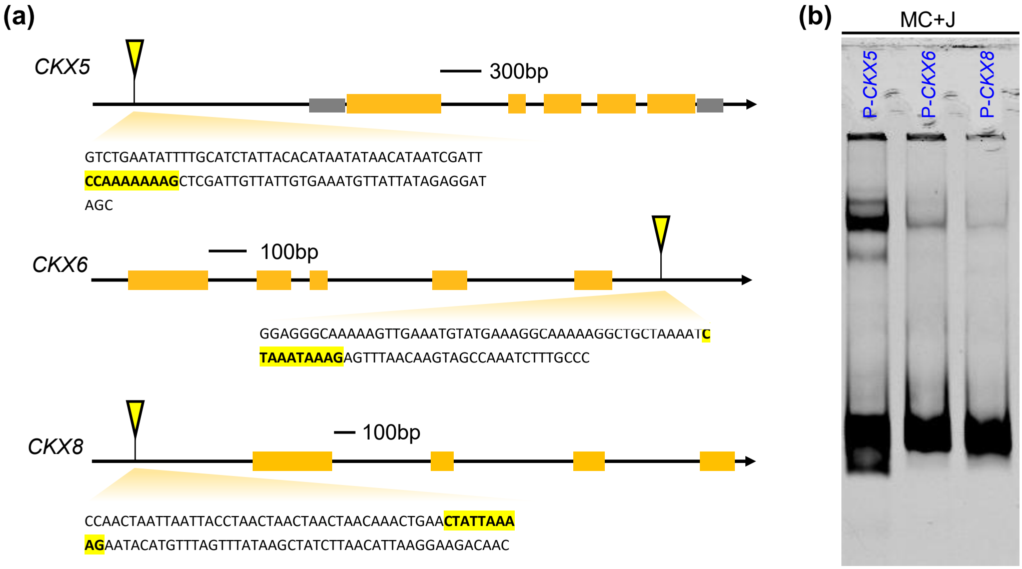

Fig S19. MC can bind to the promoters of *CKX5/6/8*. (a) Schematic representation of *CKX5/6/8* genomic loci showing CArG-box motifs. The full probe sequences of *CKX5/6/8* (P-*CKX/5/6/8*) are shown. Triangles indicate CArG boxes. (b) EMSA with MC-J and the probe fragments shown in (a)

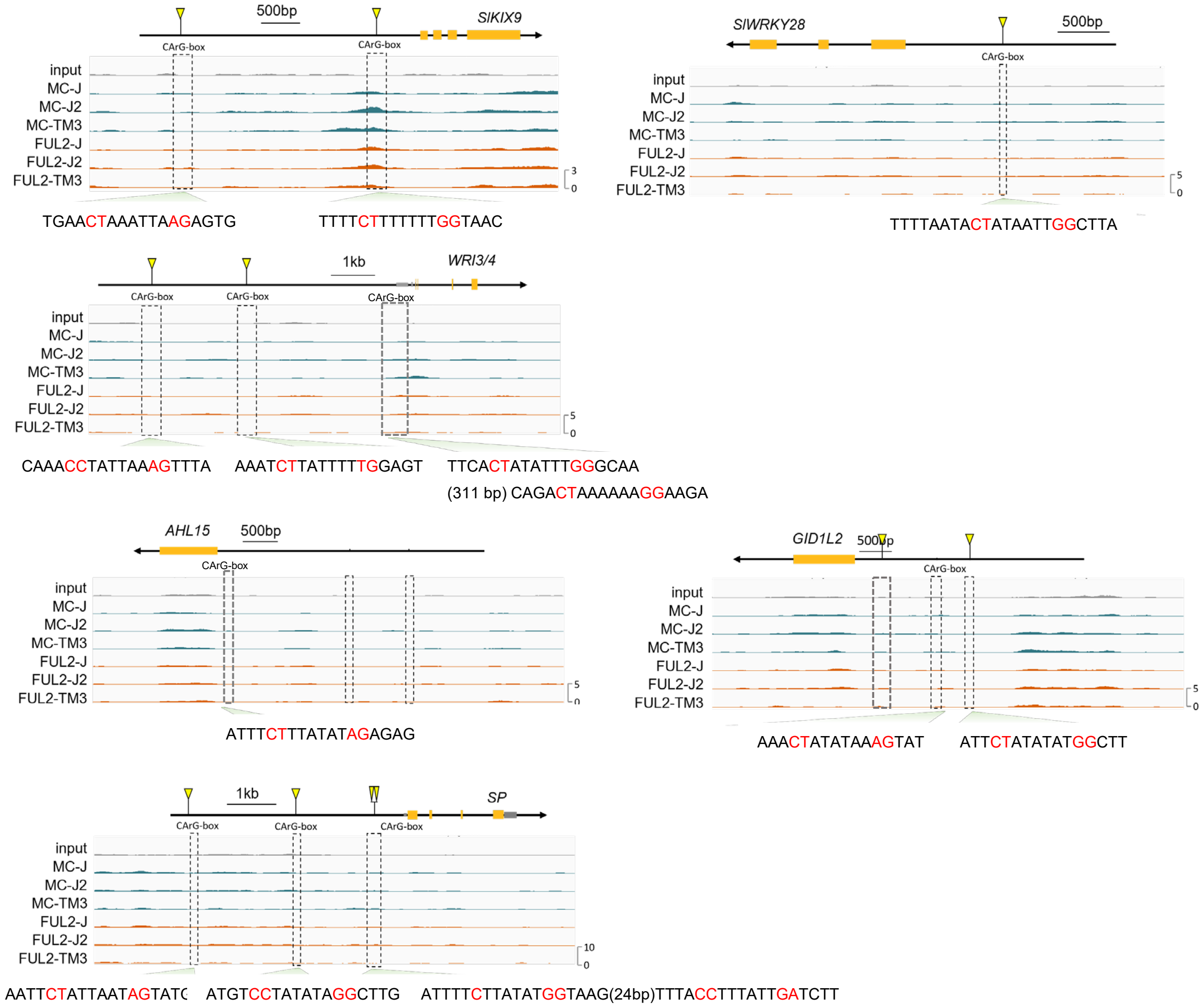

Fig. S20. Integrative Genomics Viewer (IGV) screenshots of DEGs with no significant DAP-seq peaks.

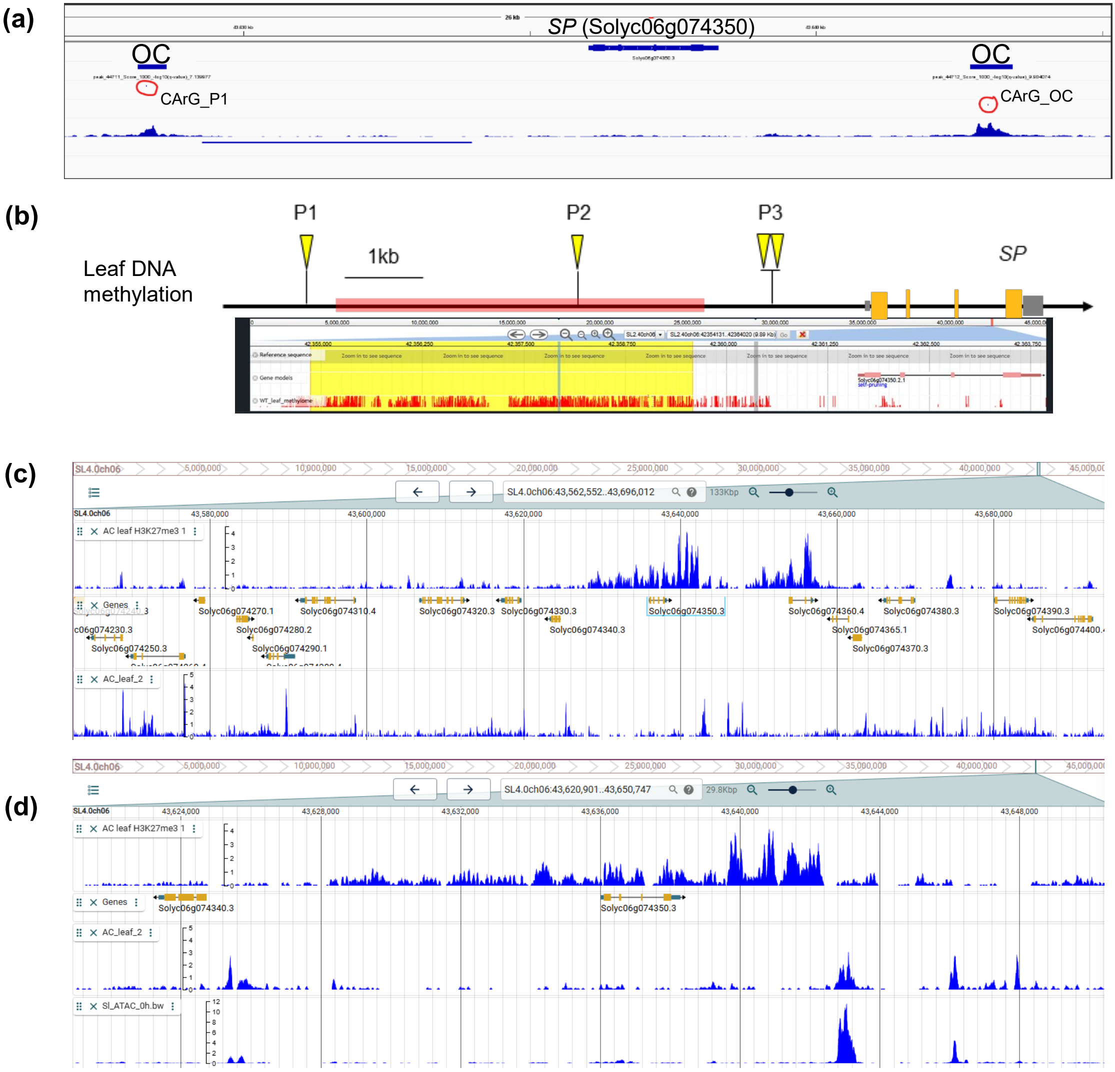

Fig S21. DNA and chromatin marks up- and downstream of *SP*. (a) In inflorescence meristem, there is a small open chromatin peak at the location of CArG-box P1 and an additional small peak at the location of the downstream CArG-box. The red circles indicate the locations of the two tested CArG-boxes. (b) Leaf DNA methylation is particularly pronounced at the TE (b), while the entire locus is in leaves silenced by H3K27me3 (c,d). The open chromatin data (Dnase-seq (middle track in (d)) and ATAC-seq (bottom track in (d)) show that the entire region is closed, except for an open chromatin peak around 5 kb 3' of the stop codon (location downstream CArG-box) (c,d). DNA methylation data from http://ted.bti.cornell.edu/epigenome/jbrowse/; other data from: http://www.epigenome.cuhk.edu.hk/jbrowse2/)

Table S1. Primers used in this study.

Oligo name	F	R	purpose
MC cloning	AAAAAAGCAGGCTTGGGAAGAGGAAAAGTTG	AGAAAGCTGGGTTCATAGATGTTTATTCATGTT	Y2H
LIN cloning	AAAAAGCAGGCTTGGGAAGAGGTAAGGTAGA	AGAAAGCTGGGTTCAAAGCATCCATCCTGGTAA	Y2H
MC-sgRNA-1	TGTGGTCTCAATTGTAACATTTTCAAAGAGAA GGTTTTAGAGCTAGAAATAGCAAG	TGTGGTCTCAAGCGTAATGCCAACTTTGTACTGT	CRISPR/Cas9
MC-sgRNA-2	TGTGGTCTCAATTGTTGAGTATTCTTCTGATTC AGTTTTAGAGCTAGAAATAGCAAG	GGTCTCAAGCGTAATGCCAACTTTGTAC	
MC genotyping	AGTGGGTTGTACTATTTCCCT	GAGACACACACTTATTTTGCAGGA	genotyping
MC promoter	GTGTGGTCTCAGGAGTATATGGAGAGAAAAA ATCCTC	ATGTGGTCTCAAGCGATTATTAAACTTAGCTCCT TTTCTACGTAGAAAAAGGAGCATTGTTTAATAAT	GUS assay
FUL2 promoter	ATGTGGTCTCAGGAGTCGACACACATCAATAC AAG	ATGTGGTCTCAAGCGATTAATTTCTTTCTTTCTTC TTTC	
FUL1 probe 1	CACATAACAAATAATCCATCTTCTCTG	CGGATATTTTTTCTATACTATGAAT	In situ hybridization
FUL1 probe 2	GATGCCACAATATATTGTCTATG	ATGGAATCATACATCAAAATTTATTT	
FUL2 probe 1	TGACAACACTTTTGACGAAACCAC	TTAATTTCTTTCTTTCTTCTTTCTTC	
FUL2 probe 2	ATTAAAACTTAATGAAGTATAAAG	GACAAAACATCCAAAGAGAGGATG	
MBP20 probe 1	ATAAAACCTCCCCCCCCAACCC	CCCATCCTCTATCTTTAATGCG	
MBP20 probe 2	ATCACATTCTCACCACCAACTTCC	CCTAGATCTTTTTAGTACTAAAAAA	
MC probe	GAGGAATAATGAGCTTGACCT	ATGAGACCACAAAATTACTAATAG	
MC	TGGACATGAATTCTTTCCTTCCTC	AGGACCAAGAACATCTGGATC	qRT-PCR
BLH1	CCAAGTACATCTCTTCCACC	GGAGTCAGGTGTCGAATTGGT	
FES1	GCTGGGGTGCATCATGTAGA	TTCCGGTTCTGGTTGCTTGA	
WRI3/4	GGCAGCTATACAGTACCGTGG	TGTTTGTGTTAGGCATGGGA	
SlKIX9	TGAGGTTCATGGCTCAGCAA	GCCTCAGAATTGGCTTTGGA	
SlWRKY28	ATGAGGTTGGAGAGCAAG	GTCTTCTCCTTCTTCTTGGCTC	
KAN2	TAGGGCTCCAAGGATGCGTT	CACCCAAGAGTTCAACAGCA	
BRC1b	GTCGCCATCAACAACAAGAG	CCTCACCCTCCGATCCCTAT	
GID1L2	CGTCGTTGCTGTTTCGGTAA	GTGCGGTCCAACAATCTTCG	
Solyc10g008410	ACATGAAGGCGGCAATCTCT	CATTGTGGGTGGCGTTGAG	
Solyc09g075680	TCTGCCAGACTCTACCTCCC	CGCCGCCGTGGTAATATACT	
Solyc07g045460	ATTGAGGGATCATGGGATGG	GATACACGCCTGATGGAAAC	
Solyc12g062200	CAGCGAGCAACATCAGCAAAC	GGAGGCGACTGTCCATGAAT	
CYP724B2	CAGTGCTGTTCATTTGGACCC	GCTCATCACTCTCCCATCTCC	
caffeoyl-CoA	GCCCTGCACTACCTGTTCTT	TGTCCTTGTCAGCATCCACA	
Solyc03g006040	GACTCCAGCTAGAAGCCATTCA	CCTACTTGTAACCCTAGC	
Solyc08g076400	TAGGGCTCCAAGGATGCGTT	CACCCAAGAGTTCAACAGCA	
SP probe 1	ATGGAATATGGAGAAAAGGG	CAGCTTTATAACTCCTACTCC	EMSA + ampDAP-qPCR
SP probe 2	TGTGCAATGTAGGAATAGTATCCCA	AGTACGACTATGGTAGGCCC	
SP probe 3	GACAAATATGATACTCCCACCGT	CTCATTTTTCTCTCTTCTCATAAATG	
SP probe OC	GAAAGAGAGATGAAAAGGG	TACTATGCTTTTAGATAACGTG	
DAP-qPCR REF1	ACCTGGAATGAGGCCTGGTG	TGTTGCTGCATCAGCGGAAC	
DAP-qPCR REF2	GGAGATTGAAACTGCCAGGAGCA	CTGCAGCTTCCATACCAATCATGG	

Table S2. FPKM values of interesting DEGs.

				WT				WT				*ful2 mbp20*			*ful2 mbp20*			*mc*			*mc*			
		**gene_id**	**Annotation**	FM_1	FM_2	FM_3	FM_4	IM_1	IM_2	IM_3	IM_4	FM_1	FM_2	FM_3	IM_1	IM_2	IM_3	FM_1	FM_2	FM_3	IM_1	IM_2	IM_3	IM_4
**commen targets**		Solyc06g068570	AP2-ERF WRINKLED ortholog	0.26	0.00	0.05	0.04	0.07	0.03	0.00	0.00	6.08	4.48	4.46	8.88	7.38	7.70	6.80	6.14	5.71	8.08	7.07	7.87	9.28
		Solyc08g059700	SlKIX9	0.86	0.11	0.51	0.13	0.30	0.11	0.13	0.00	21.30	20.06	18.48	22.42	18.70	25.43	29.12	20.91	26.82	29.80	23.74	16.67	29.47
		Solyc04g064770	FES1-homolog (zinc-finger TF)	10.60	1.77	4.65	3.92	8.31	4.07	12.50	5.13	47.31	51.11	51.95	53.72	53.52	55.73	61.12	63.05	57.78	57.48	60.24	58.26	61.46
		Solyc12g011200	SlWRKY28	1.20	0.24	0.45	0.44	0.31	0.25	0.31	0.25	4.82	4.00	5.03	5.18	6.01	5.29	7.17	5.91	6.58	6.79	6.09	5.32	9.01
		Solyc12g087950	AHL15-homolog	0.67	0.96	0.79	0.54	0.70	0.51	0.41	0.45	2.13	2.28	2.67	2.55	1.93	2.63	2.40	2.35	2.68	2.31	1.79	2.66	2.17
		Solyc01g007070	BLH1	0.54	0.31	0.34	0.15	0.21	0.20	0.16	0.22	1.32	0.86	1.55	1.98	1.63	2.08	1.90	1.07	2.72	1.42	2.04	1.19	2.43
**MC specific targets**	IM/FM	Solyc12g077630	Unknown Protein	0.95	1.14	1.50	1.07	1.06	1.41	1.21	1.30	0.85	0.58	2.18	1.35	1.59	0.91	2.47	5.03	3.50	4.80	4.27	4.44	3.36
	FM	Solyc12g077620	Unknown Protein	1.62	0.86	0.59	0.72	0.91	0.82	1.41	1.02	0.84	0.68	1.35	0.76	2.06	1.61	3.23	4.69	3.64	3.21	3.49	3.10	2.42
	FM	Solyc10g008410	RING finger protein 5	12.54	5.75	10.25	8.16	8.46	7.52	16.78	9.69	20.41	13.36	11.11	21.11	29.67	32.82	58.97	29.79	45.56	46.79	43.31	24.20	38.15
	IM	Solyc09g098050	Ankyrin repeat-containing protein At5g02620	0.46	0.44	0.65	0.28	0.66	0.86	0.54	0.60	0.31	0.48	0.47	0.56	0.61	0.65	1.47	1.06	0.93	2.45	1.70	1.56	1.94
	FM	Solyc09g084460	Chymotrypsin inhibitor 2	0.99	0.24	0.83	0.53	0.40	0.83	0.34	0.78	0.61	0.85	0.78	0.51	0.78	0.76	3.32	3.06	1.85	1.82	1.01	0.84	1.18
	FM	Solyc08g076400	Myb family transcription factor-like	5.19	3.78	4.93	4.91	9.64	7.59	6.50	9.81	5.57	5.42	3.49	8.40	5.81	8.62	13.52	9.92	9.78	14.89	11.19	10.59	14.57
	FM	Solyc07g056160	CYP724B2 mRNA for cytochrome P450	42.49	32.39	46.59	32.45	38.58	36.87	32.39	40.22	61.76	46.53	37.05	48.95	49.93	39.71	132.60	93.50	119.60	102.42	73.04	86.77	117.86
	FM	Solyc07g052220	Unknown Protein	1.39	1.55	0.39	1.47	1.87	1.18	0.45	1.30	1.66	0.88	1.50	2.26	1.84	2.73	3.72	4.97	3.58	2.65	4.79	4.49	3.03
	FM	Solyc07g045460	5-AMP-activated protein kinase subunit beta-1	6.15	4.71	4.43	4.72	12.05	8.29	9.07	10.05	6.89	5.78	4.83	12.81	10.83	15.34	18.24	9.48	13.43	17.14	15.50	11.99	19.44
	FM	Solyc06g074350	self-pruning	0.23	0.23	0.27	0.47	3.70	2.69	15.83	0.48	1.39	0.51	0.07	2.73	3.71	0.45	13.04	4.63	5.48	19.32	26.73	19.89	10.37
	FM	Solyc06g005000	IBR finger domain-containing protein	6.49	5.43	8.72	9.77	7.63	9.19	10.31	9.08	7.12	7.25	7.29	8.06	3.37	10.19	3.40	2.27	1.19	2.67	3.19	2.23	2.00
	IM	Solyc05g056620	Macrocalyx	162.64	166.47	201.29	169.57	181.55	156.97	161.65	171.93	195.59	193.74	188.29	184.55	163.26	163.75	65.12	75.01	73.58	61.22	55.61	60.18	61.84
	IM	Solyc05g021510	Unknown Protein	5.00	6.24	5.72	4.03	5.77	7.42	6.69	6.51	1.53	2.68	4.56	3.62	1.90	3.77	2.70	3.10	3.81	2.86	3.12	2.89	3.64
	FM	Solyc04g076037	orphan gene	0.00	0.00	0.00	0.00	0.00	0.77	21.94	0.00	0.00	0.00	0.00	0.92	0.00	0.00	24.78	23.92	1.00	31.66	27.71	0.00	0.00
	IM	Solyc04g014510	glutamine synthase	4.80	3.68	6.01	2.89	5.69	6.92	6.25	5.08	4.63	3.79	3.33	5.99	6.29	5.00	4.96	3.77	2.03	2.31	2.79	2.05	3.49
	FM	Solyc03g117330	Prolyl-tRNA synthetase	5.66	5.25	5.84	5.34	5.97	5.60	6.05	5.14	8.25	5.34	5.12	5.96	8.56	6.83	17.98	14.98	12.13	16.61	13.87	15.78	15.24
	IM	Solyc03g078210	Unknown Protein	0.75	1.59	1.43	1.63	1.52	1.38	1.84	1.63	1.52	0.90	1.34	1.58	1.47	1.36	0.45	0.34	0.98	0.31	0.40	0.45	0.76
	IM	Solyc03g006040	DNA binding protein	28.93	23.21	20.46	34.63	30.49	25.67	19.46	32.44	22.03	21.44	14.68	25.11	21.54	38.96	63.48	30.62	38.15	77.36	72.54	38.41	51.65
	FM	Solyc02g081390	Amine oxidase family protein	2.30	1.82	1.52	2.00	2.23	2.08	2.52	1.75	1.83	1.88	1.77	1.82	2.70	3.33	6.13	3.42	3.78	6.05	3.59	3.39	5.27
	IM	Solyc02g069420	Phospholipid-translocating P-type ATPase flippase	0.52	0.98	2.14	0.50	2.60	2.97	3.26	3.34	2.58	1.81	2.97	2.71	3.96	1.69	1.74	1.97	1.64	2.07	0.47	1.47	0.97
	IM	Solyc02g068140	Nodulin-like protein	1.66	1.08	6.27	0.57	4.64	6.39	5.54	3.57	6.36	3.80	1.03	5.00	5.06	3.64	1.16	5.67	1.26	2.02	1.56	3.76	2.41
**FUL2/MBP20 specific targets**	FM	Solyc01g056657	Unknown protein	0.00	0.00	0.15	0.00	0.00	0.00	0.00	0.00	0.00	0.00	0.00	0.00	0.00	0.00	0.00	1.74	2.96	2.57	2.74	1.64	1.94
	IM	Solyc01g067255	Unknown protein	1.11	1.29	0.87	0.70	0.90	0.90	1.20	0.81	0.76	0.56	1.14	0.71	0.99	0.49	1.19	2.36	2.05	1.44	2.12	2.21	2.29
	FM/IM	Solyc01g081480	GPI inositol-deacylase PGAP1-like protein	1.08	1.35	0.94	1.21	1.28	1.42	0.52	0.90	3.62	4.40	3.78	4.08	2.96	3.21	0.64	0.65	1.61	1.15	0.83	0.50	0.74
	IM	Solyc01g108050	Dynamin-related protein 3B	1.34	1.06	1.34	2.26	1.16	1.88	1.38	1.51	2.39	3.14	2.54	2.43	3.23	3.72	1.43	1.02	1.38	1.32	1.00	0.97	1.07
	IM	Solyc02g087800	Aldose-1-epimerase-like protein	0.55	0.42	0.36	0.49	0.88	0.67	0.74	0.55	0.62	0.72	0.75	1.34	1.53	1.88	0.30	0.35	0.68	0.58	0.73	0.60	0.77
	IM	Solyc02g093270	caffeoyl-CoA O-methyltransferase	13.64	10.66	12.60	11.35	10.46	14.99	22.77	14.95	26.90	32.57	29.18	31.30	41.57	34.45	13.37	14.05	16.01	15.50	18.86	8.79	21.62
	IM	Solyc04g077690	Unknown Protein	4.74	5.27	3.93	4.70	4.71	6.94	5.08	5.96	5.73	7.86	13.72	12.69	9.39	12.89	5.22	4.77	4.44	6.46	6.48	5.14	4.83
	FM	Solyc04g081700	Unknown Protein	0.00	0.09	0.07	0.09	0.00	0.14	0.00	0.00	7.59	0.36	2.90	0.00	0.00	0.00	0.00	0.00	0.00	0.00	0.11	0.00	0.00
	IM	Solyc05g055020	Light-dependent short hypocotyls 1	1.21	1.75	1.13	2.17	0.99	1.26	1.12	0.98	1.05	1.53	1.46	1.84	1.86	3.10	1.07	0.61	1.43	0.63	1.29	0.83	0.80
	FM	Solyc06g008820	Na+/H+ antiporter 1	3.34	3.36	3.42	3.01	1.79	2.25	2.31	2.80	8.04	8.08	8.19	7.32	8.92	8.46	3.18	3.46	3.81	3.48	3.27	3.20	4.09
	FM	Solyc06g049033	Unknown protein	0.13	0.00	0.00	0.00	0.00	0.00	0.00	0.00	3.37	3.52	3.65	3.64	4.07	3.56	0.00	0.00	0.00	0.00	0.00	0.00	0.00
	IM	Solyc06g050280	CBL-interacting protein kinase 13	1.11	0.98	1.25	1.12	1.20	1.81	1.55	1.65	3.20	4.60	2.97	5.11	4.32	4.52	1.11	1.61	1.31	1.03	2.15	1.73	1.18
	FM/IM	Solyc06g050290	CBL-interacting protein kinase 13	1.06	0.81	0.79	1.13	0.68	1.04	0.75	1.14	2.26	2.27	2.00	2.06	1.89	2.28	0.59	0.57	1.38	0.45	0.95	0.94	0.94
	IM	Solyc07g150147	Unknown protein	0.67	0.63	0.59	1.26	1.77	2.00	0.98	1.47	0.91	1.06	0.59	3.15	2.77	5.58	0.75	0.18	0.96	1.12	1.73	1.24	1.10
	IM	Solyc09g062960	Unknown Protein	0.40	0.00	0.00	0.00	0.00	0.00	0.07	0.00	0.84	2.25	0.09	5.82	8.55	0.15	0.00	0.03	0.00	0.00	0.00	0.00	0.00
	FM/IM	Solyc09g075680	Gibberellin receptor GID1L2	2.29	1.84	3.17	2.36	2.52	1.88	1.23	1.94	6.10	4.47	4.53	5.69	4.68	5.74	3.50	2.08	1.90	1.82	1.51	1.92	2.40
	IM	Solyc09g082190	Unknown Protein	0.46	0.99	2.33	0.14	1.27	2.29	1.18	1.24	4.09	3.42	4.07	3.58	3.08	7.71	1.05	0.26	0.53	1.04	0.12	0.51	0.07
	FM	Solyc11g006740	F-box protein PP2-B1	1.12	0.82	1.43	0.98	0.84	1.34	1.25	0.80	2.51	2.57	2.17	2.20	2.99	2.62	1.00	1.02	1.20	1.24	0.94	1.53	1.44
	FM	Solyc12g062200	Orphan gene, unknown protein	2.55	0.12	0.00	0.08	0.16	0.00	0.10	0.15	63.45	72.01	73.66	64.39	67.39	70.93	0.00	0.16	0.32	0.12	0.10	1.18	0.25

Table S3. Tomato gene accession numbers.

Gene Name	Gene ID	Gene Name	Gene ID
FUL1	Solyc06g069430	*MBP24*	Solyc01g105800
FUL2	Solyc03g114830	*MBP13*	Solyc08g080100
MBP10	Solyc02g065730	*MBP14*	Solyc12g056460
MBP20	Solyc02g089210	*MBP9*	Solyc04g076680
MC	Solyc05g056620	*MBP12*	Solyc12g088090
J	Solyc11g010570	*MBP22*	Solyc11g005120
J2	Solyc12g038510	*SP*	Solyc06g074350
EJ2	Solyc03g114840	*SPGB*	Solyc02g083520
TM29	Solyc02g089200	*CKX5*	Solyc04g016430
MADS-RIN	Solyc05g012020	*CKX6*	Solyc12g008900
TM5	Solyc05g015750	*CKX8*	Solyc10g017990
TM3	Solyc01g093965	*AHL15-like*	Solyc12g087950
STM3	Solyc01g092950	*FES1-like*	Solyc04g064770
MBP18	Solyc03g006830	*WRI3/4-like*	Solyc06g068570
TAG1	Solyc02g071730	*KIX9-like*	Solyc08g059700
MBP24	Solyc01g105800	*SlWRKY28*	Solyc12g011200
BLH1	Solyc01g007070	*AP2a*	Solyc03g044300
CYP724B2	Solyc07g056160	*AP2b*	Solyc02g064960
KAN2	Solyc08g076400	*AP2c*	Solyc02g093150
BRC1b	Solyc02g089830	*GID1L2*	Solyc09g075680
caffeoyl-CoA	Solyc02g093270	*RMA1-like*	Solyc10g008410
SPL15	Solyc10g078700		

References:

Bemer M, van Mourik H, Muiño JM, Ferrándiz C, Kaufmann K, Angenent GC. 2017. FRUITFULL controls SAUR10 expression and regulates Arabidopsis growth and architecture. *Journal of Experimental Botany* **68**(13): 3391-3403.

Jiang X, Lubini G, Hernandes-Lopes J, Rijnsburger K, Veltkamp V, de Maagd RA, Angenent GC, Bemer M. 2022. FRUITFULL-like genes regulate flowering time and inflorescence architecture in tomato. *The Plant Cell* **34**(3): 1002-1019.

Smaczniak C, Immink RG, Angenent GC, Kaufmann K. Developmental and evolutionary diversity of plant MADS-domain factors: insights from recent studies. Development. 2012 Sep;139(17):3081-98. doi: 10.1242/dev.074674. PMID: 22872082.

Wang Y, Zhang J, Hu Z, Guo X, Tian S, Chen G. Genome-Wide Analysis of the MADS-Box Transcription Factor Family in *Solanum lycopersicum*. Int J Mol Sci. 2019 Jun 18;20(12):2961. doi: 10.3390/ijms20122961. PMID: 31216621; PMCID: PMC6627509.

Zahn IE, Roelofsen C, Angenent GC, Bemer M. 2023. TM3 and STM3 Promote Flowering Together with FUL2 and MBP20, but Act Antagonistically in Inflorescence Branching in Tomato. *Plants* **12**(15): 2754.
